# *Staphylococcus aureus* Exploits a Non-ribosomal Cyclic Dipeptide to Modulate Survival within Epithelial Cells and Phagocytes

**DOI:** 10.1371/journal.ppat.1005857

**Published:** 2016-09-15

**Authors:** Sebastian Blättner, Sudip Das, Kerstin Paprotka, Ursula Eilers, Markus Krischke, Dorothee Kretschmer, Christian W. Remmele, Marcus Dittrich, Tobias Müller, Christina Schuelein-Voelk, Tobias Hertlein, Martin J. Mueller, Bruno Huettel, Richard Reinhardt, Knut Ohlsen, Thomas Rudel, Martin J. Fraunholz

**Affiliations:** 1 Biocenter, Chair of Microbiology, University of Würzburg, Würzburg, Germany; 2 Core Unit Functional Genomics, University of Würzburg, Würzburg, Germany; 3 Biocenter, Chair of Pharmaceutical Biology, University of Würzburg, Würzburg, Germany; 4 Department of Infection Biology, Interfaculty Institute for Microbiology and Infection Medicine Tübingen (IMIT), University Tübingen, Tübingen, Germany; 5 Biocenter, Chair of Bioinformatics, University of Würzburg, Würzburg, Germany; 6 Institute of Human Genetics, University of Würzburg, Würzburg, Germany; 7 Institute for Molecular Infection Biology, University of Würzburg, Würzburg, Germany; 8 Max Planck Genome Centre, Cologne, Germany; Columbia University, UNITED STATES

## Abstract

Community-acquired (CA) *Staphylococcus aureus* cause various diseases even in healthy individuals. Enhanced virulence of CA-strains is partly attributed to increased production of toxins such as phenol-soluble modulins (PSM). The pathogen is internalized efficiently by mammalian host cells and intracellular *S*. *aureus* has recently been shown to contribute to disease. Upon internalization, cytotoxic *S*. *aureus* strains can disrupt phagosomal membranes and kill host cells in a PSM-dependent manner. However, PSM are not sufficient for these processes. Here we screened for factors required for intracellular *S*. *aureus* virulence. We infected escape reporter host cells with strains from an established transposon mutant library and detected phagosomal escape rates using automated microscopy. We thereby, among other factors, identified a non-ribosomal peptide synthetase (NRPS) to be required for efficient phagosomal escape and intracellular survival of *S*. *aureus* as well as induction of host cell death. By genetic complementation as well as supplementation with the synthetic NRPS product, the cyclic dipeptide phevalin, wild-type phenotypes were restored. We further demonstrate that the NRPS is contributing to virulence in a mouse pneumonia model. Together, our data illustrate a hitherto unrecognized function of the *S*. *aureus* NRPS and its dipeptide product during *S*. *aureus* infection.

## Author Summary

*Staphylococcus aureus* is a notorious microbe that causes a variety of diseases in man ranging from skin abscesses to blood poisoning. *S*. *aureus* can be internalized by cells of its human host, break out of membrane enclosures, and subsequently kill the cells from within. For these processes the bacterium usually employs an arsenal of partially unknown toxic proteins and peptides. In a search for factors that enable *S*. *aureus* to survive inside host cells we employed automated fluorescence microscopy. We thereby found that *S*. *aureus* uses a small cyclic dipeptide, phevalin, to interfere with host processes that would lead to destruction of the pathogen. Phevalin is not formed by ribosomes, but by a multi-domain peptide assembly line. Whereas such non-ribosomal peptides often act as antibiotics or in interspecies competition, we show that the production of phevalin not only enhances intracellular survival of *S*. *aureus* and increases killing of host cells, but also is involved in lung infection. Since we show that phevalin targets the host cell, this also implies signaling across the domains of life—between bacteria and human cells.

## Introduction

*Staphylococcus aureus* is a notorious human pathogen that can cause a variety of diseases and thus causes a dramatic disease burden and death toll especially in the hospital setting [1,2]. The advent of methicillin resistant *S*. *aureus* (MRSA) led to a loss of therapeutic options and in recent years a shift was observed from mostly healthcare-associated (HA) to community-acquired (CA) MRSA infections. CA-MRSA strains are epidemic and often cause serious infections in otherwise healthy individuals. Enhanced virulence of CA-MRSA has been attributed in part to increased production levels of toxins such as phenol-soluble modulins (PSM)[3,4,5].

Studies in recent years have shown that *S*. *aureus* is a facultative intracellular pathogen since it is not only internalized by professional but also by non-professional phagocytes (e.g. reviewed in [6]). Further, intracellularity has recently been shown to foster dissemination of *S*. *aureus* in disease models [7]. Following internalization *S*. *aureus* is capable of avoiding destruction within the phagolysosome by disruption of the endosomal membranes [8,9,10,11,12]. *S*. *aureus* disrupts phagosomal membranes using PSMs [9,12] and readily kills host cells in a PSM-dependent manner [13,14,15]. However, PSMs are not sufficient to mediate these processes [12]. Further, in phagocytes *S*. *aureus* has been reported to grow inside mature phagolysosomes and is able to kill host cells without prior translocation to the host cytoplasm [16,17].

In order to identify additional genes involved in intracellular survival of *S*. *aureus*, we sought to identify MRSA mutants within an established transposon mutant library [18] that were not able to escape the phagosome upon internalization by host cells. We detected phagosomal escape rates by automated fluorescence microscopy using transgenic escape reporter cells. These cells express a fluorescent marker which is recruited to the staphylococcal cell wall once the bacteria are located within the host cell cytoplasm [9,12]. Among others, we thereby identified two genes, *aus*A and B, that were required for efficient escape. Both genes are arranged in a bicistronic operon and encode subunits of the non-ribosomal peptide synthase (NRPS) AusA and a phosphopantetheinyl transferase AusB, which is involved in AusA activation[19]. An initially described virulence-regulatory function of the NRPS [20] was later found to be caused by a secondary site mutation and NRPS involvement in virulence was contested [21]. The NRPS AusAB produces three cyclic dipeptides named phevalin, tyrvalin, and leuvalin [19] which belong to the class of monoketopiperazines or pyrazinones. Non-ribosomally synthesized peptides are widely distributed among a variety of bacteria species, where they assume functions as antibiotics, siderophores or toxins [22,23,24]. Notably, the major product of the NRPS, phevalin, was initially identified in *Streptomyces spp*. in a screen for calpain inhibitors [25]. While the NRPS/phevalin function in staphylococcal virulence is debated [20,21], it has been demonstrated to exert effects on host cells. In keratinocytes, addition of pure phevalin or staphylococcal supernatants containing the substance resulted in transcriptional changes which illustrate that a host cell target of the cyclic dipeptide exists [26].

Here we show that supplementation with the cyclic dipeptide phevalin[20,25] as well as genetic complementation of a insertional mutant of *aus*B restored escape in epithelial cell lines and intracellular cytotoxicity of MRSA in epithelial cells as well as human and murine primary macrophages, and that phevalin plays a role in in a pneumonia infection model. Together, our data illustrate a hitherto unrecognized function of the *S*. *aureus* NRPS AusAB and its dipeptide product, phevalin, during intracellular *S*. *aureus* infection, host-pathogen interaction and in a mouse pneumonia model.

## Results and Discussion

**Fig 1:**
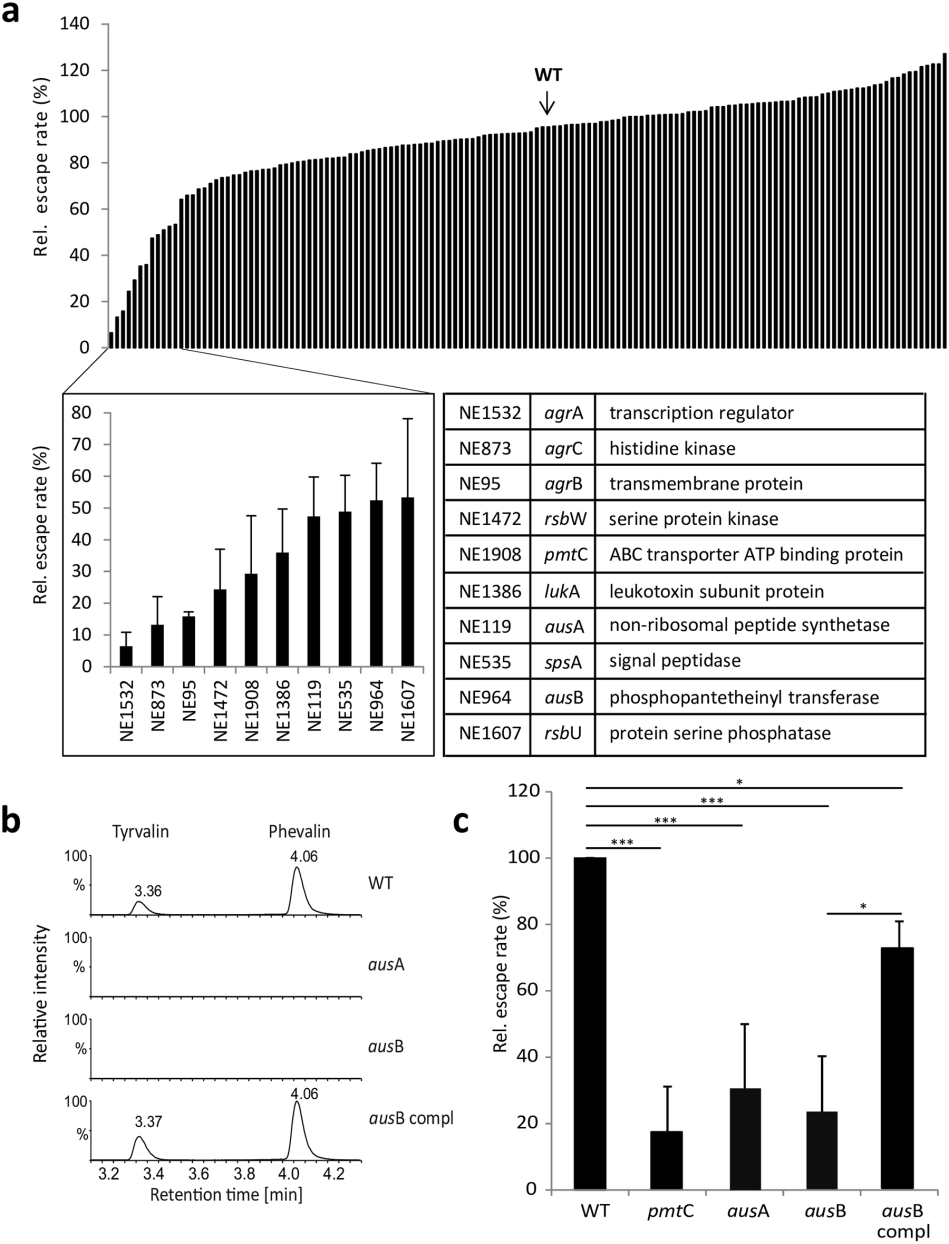
A screen for *S*. *aureus* phagosomal escape phenotypes identifies a non-ribosomal peptide synthethase AusAB. **a**) HeLa YFP-CWT escape reporter cells were infected with *S*. *aureus* JE2 mutants at a multiplicity of infection of 10. After 4 hours cells were fixed and phagosomal escape was determined using automated fluorescence microscopy. Wild type infection was normalized to 100%. Whereas many mutants showed proficiency in phagosomal escape within a window of ±20% relative to the wild type (WT, arrow), several mutants were severely affected in their ability to translocate to the host cell cytoplasm (for details see S4 Table). **b**) UPLC-MS detection of aureusimines in stationary phase culture supernatants of *S*. *aureus*. Production of aureusimine A (tyrvalin) and B (phevalin) is lost in the *S*. *aureus aus*A and *aus*B mutants, but is readily detected in wild type (WT) and complemented *aus*B mutant. Y-axis shows relative intensity (per cent). X-axis: retention time (min). Chromatograms display the combination of three multiple reaction monitoring (MRM) measurements each for tyrvalin and phevalin, respectively, and Y-axes were fixed for the highest signal throughout the samples to allow for comparison of intensities. **c**) Escape rates of mutants within *aus*A and *aus*B are reduced to levels of the negative control, *pmt*C, a mutant in the PSM transporter. Genetic complementation (*aus*B compl) of the escape phenotype within the *aus*B mutant was observed. Bar graphs show the mean of three independent experiments ± SD. Statistical analysis was performed by t-test. *P<0.05; ***P<0.001.

We assessed phagosomal escape of *S*. *aureus* with a transgenic HeLa cell line expressing a fluorescent reporter protein, YFP-CWT, which results in the recruitment of the cytoplasmic fluorophore to the staphylococcal cell wall once the endosomal membrane barrier is breached[9,12,27]. Single gene mutants in the CA-MRSA strain JE2 were obtained from the Nebraska transposon mutant library[18]. We infected the reporter cell line with fluorescently labeled bacteria in multi-well dishes. Four hours post-infection we fixed the samples and compared escape rates of each mutant with that of wild type bacteria by automated microscopy (S1a Fig). We investigated relative escape rates for a set of mutants in known and potential virulence factors and regulators (S4 Table). Whereas most mutants demonstrated only small differences in escape (±20% of the wild-type), we identified several that showed a drastic decrease in phagosomal escape four hours post-infection (Fig 1a). As expected, these included mutants within the quorum sensing system genes *agr*A, B, and C (relative escape rates: 6.4 ± 4.4%, 15.8 ± 1.4%, and 13.2 ± 8.8%, respectively), as well as the PSM transporter gene *pmt*C (29.3± 18.3%). Similarly, *rsb*U and *rsb*W (53.3 ± 24.8% and 25.6 ± 9.2%, respectively), whose products are involved in regulation of the alternative sigma factor σB, the non-functional homolog of type I signal peptidase *sps*A (48.8 ± 11.5%), as well as the leukocidin subunit *luk*A (35.9 ± 13.7%) demonstrated involvement in phagosomal escape (Fig 1a and S4 Table). *luk*A encodes a subunit of the pore-forming toxin (PFT) LukAB (LukGH), which has been implicated in neutrophil cell death[15,28], thus may also possess an intracellular target in epithelial cells. *rsb*U and *rsb*W[29] are modulators of the alternative sigma factor σB, which was shown to be required for adaptation of *S*. *aureus* to an intracellular environment[30]. SpsA is known as “non-functional”type I signal peptidase[31], although our assay demonstrates a contribution of SpsA to phagosomal escape. Further experiments will be required to determine the mode of action of LukA and SpsA.

Interestingly, both genes of the *aus*AB operon, which encodes a non-ribosomal peptide synthetase (NRPS)[19] were also identified in our screen. For the *aus*A and *aus*B mutant relative phagosomal escape rates were reduced to 47.3 ± 12.4% and 52.4 ± 11.7%, respectively (Fig 1a). Since the contribution of aureusimines to *S*. *aureus*-induced disease is debated [20,21], we more closely investigated the *aus*AB operon. By Ultra Performance Liquid Chromatography (UPLC) we demonstrated the absence of NRPS products, aureusimine A (tyrvalin) and B (phevalin), in bacterial culture supernatants of *aus*A and *aus*B mutants, respectively (Fig 1b and S2a Fig). A plasmid constitutively expressing the AusB phosphopantetheinyl transferase complemented the *aus*B mutant as evidenced by the production of the NRPS products, tyrvalin and phevalin (Fig 1b), as well as restoration of the escape phenotype in HeLa (Fig 1c). These observations were independent of the host cell type used, since the same results were obtained within an upper airway epithelial cell line (S1c Fig).

**Fig 2:**
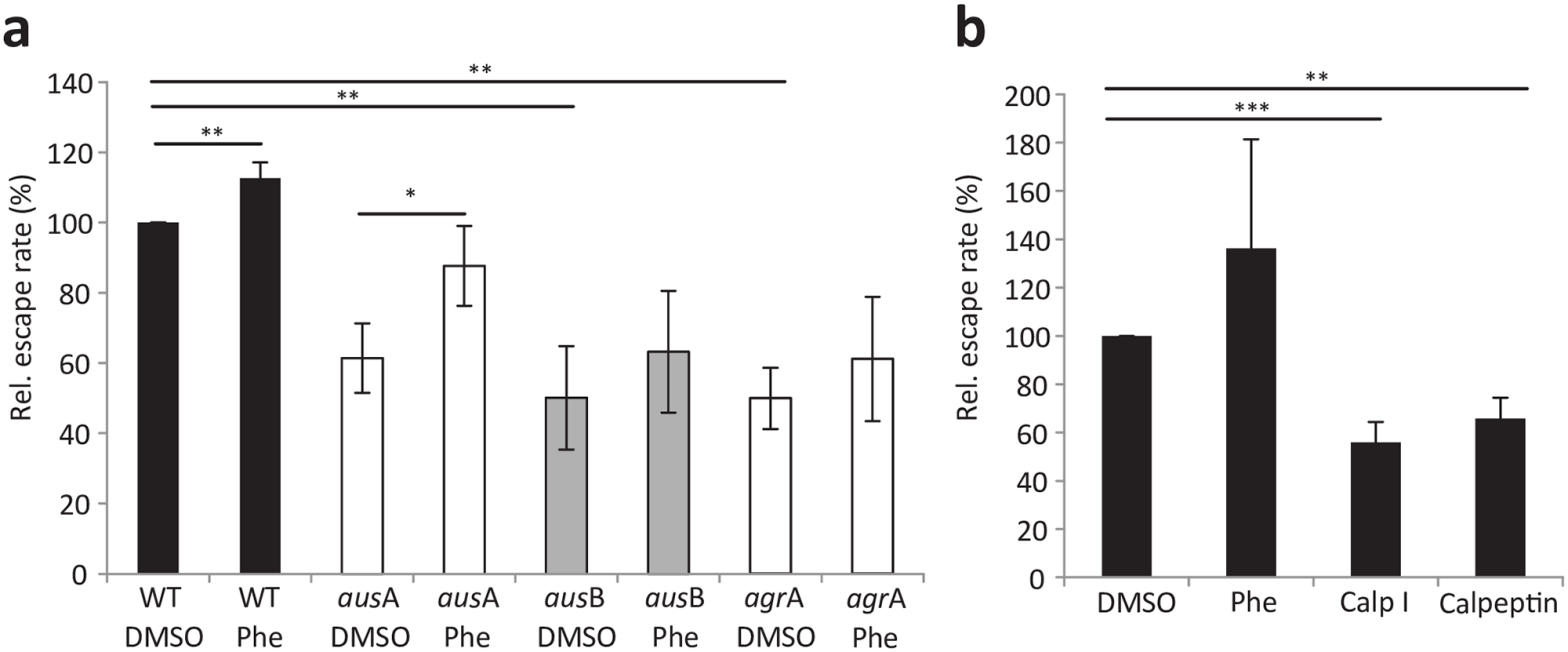
Synthetic phevalin increases phagosomal escape rates of *S*. *aureus*. **a**) At the time of infection, 10 μM phevalin was added simultaneous with *S*. *aureus* JE2 wild type (WT) or mutant strains to host cells. Concentration-dependent increase in phagosomal escape rates of bacteria was observed in the phevalin-treated samples when compared to solvent control. **b**) Phevalin does not act as calpain inhibitor. During infection with *S*. *aureus* (WT), host cells were either treated with 10 μM phevalin (Phe), calpain inhibitor I (Calp I), Calpeptin, or solvent control (DMSO). Whereas Calp I and Calpeptin caused a significant reduction of *S*. *aureus* phagosomal escape, phevalin did not, thereby suggesting a different mode of action of the pyrazinone. Bar graphs show the mean of three independent experiments ± SD. Statistical analysis was performed by t-test. *P<0.05; **P<0.01; ***P<0.001.

Next, we determined that external addition of synthetic phevalin to tissue culture medium restored escape of intracellular *aus* mutants (Fig 2a). Interestingly, escape rates of the wild type were also enhanced by supplementation of external phevalin (Fig 2a). Externally added phevalin was readily detected by UPLC after thorough washing of host cells, thus demonstrating its cellular association (S2b Fig). Moreover, we detected phevalin produced by intracellular wild type bacteria four hours after infection (S2c Fig). Transcription analysis of the *aus* operon corroborated the *agr* dependency of *aus*A [32]. By contrast, *aus*B, seems to be *agr*-independently up-regulated during stationary phase (*P* = 0.04) suggesting alternative transcription initiation sites might exists (S6 Fig) [33].

Phevalin treatment of host cells does not cause non-specific rupture of endosomes, since formaldehyde-fixed *S*. *aureus* did not translocate to the cytoplasm of reporter cells, regardless of phevalin supplementation (S3a Fig). Thus our data demonstrate that phevalin enhances phagosomal escape of viable *S*. *aureus*, compatible with the observation that toxins, such as phenol-soluble modulins, are required for translocation of the bacteria to the host cell cytosol.

Phevalin was initially discovered in a screen for calpain inhibitors in a pool of natural products of *Streptomyces*[25]. Calpains, a family of calcium-activated cysteine proteases ubiquitously present in eukaryotic cells, are known to modulate lysosomal integrity[34]. We therefore investigated, if phevalin activity in phagosomal escape is connected to an inhibition of host cell calpains. We compared Calpain inhibitor I (Calp I) and Calpeptin to phevalin and solvent control in phagosomal escape assays. Whereas Calp I and Calpeptin significantly reduced phagosomal escape of *S*. *aureus*, phevalin treatment rather increased the rate of phagosomal escape (Fig 2b). This indicated *i*) an involvement of the host calpain proteases in translocation of *S*. *aureus* to the cytoplasm and *ii*) a mode of action for phevalin distinct from calpain inhibition.

**Fig 3:**
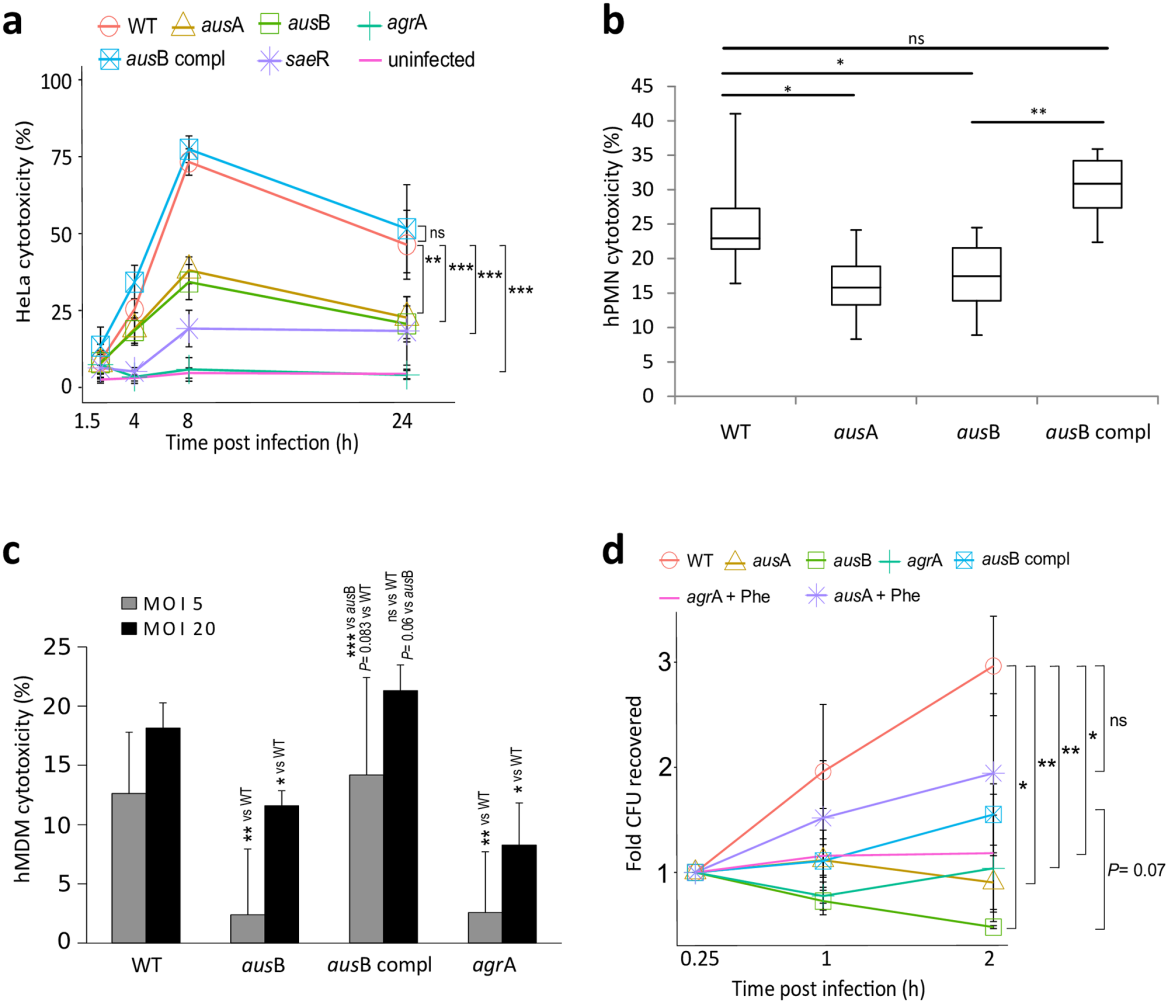
*S*. *aureus aus* mutants show altered virulence in epithelial cells and human neutrophils. **a**) HeLa cells were left uninfected (control) or were infected with *S*. *aureus* JE2 wild type (WT), isogenic *aus*A and *aus*B mutants, a complemented *aus*B mutant (*aus*B compl), or mutants within the virulence regulators *agr*A and *sae*R. Kinetics of cytotoxicity was monitored over time (x-axis) by propidium iodide (PI) staining and flow cytometry (y-axis; HeLa cytotoxicity in %). The *agr*A mutant did not demonstrate detectable host cell death. *sae*R, *aus*A and *aus*B mutants were significantly diminished in their ability to kill host cells. Genetic complementation of *aus*B recovered the phenotype of the insertional mutant. The apparent reduction of cytotoxicity at 24 hours p.i. is mediated by extended fragmentation of cells, which therefore are outside of the FSC-SSC gate for viable cells. Statistical analysis was performed by linear modeling and ANOVA followed by Tukey’s post-hoc analysis; ***P < 0.001 (Wt vs. *aus*A or *aus*B; 8 h p.i.). Host cell death rates are reduced in (**b**) hPMN 4 hours p.i. or(**c**) primary human monocyte-derived macrophages (hMDM) 2 hours p.i. when the phagocytes were infected with *aus* mutants. Cell death was measured by LDH release as percentage relative to the complete lysate (positive control) and supernatant of uninfected cells (set to 0% cell death, negative control). **d**) The NRPS AusAB is involved in bacterial survival upon macrophage co-incubation. CFUs of wild type (WT), insertional mutants within *aus*A, *aus*B and *agr*A were recovered at the indicated time points and numbers were normalized to the 15 min time point. Phevalin (+Phe) was added to selected samples at a concentration of 10 μM to assess complementation with the synthetic dipeptide. Graphs show the mean of at least three independent experiments ± SD. Statistical analyses were performed by t-test (b,c) and Wilcoxon rank sum test (d). *P < 0.05; **P<0.01; ***P < 0.001.

Phagosomal escape of *S*. *aureus* is intimately linked to subsequent induction of host cell death in epithelial cells [12]. We therefore infected epithelial cells with *S*. *aureus* wild type, isogenic *aus*A and *aus*B mutants, a complemented *aus*B mutant, as well as mutants within the global regulators *agr*A and *sae*R and compared their kinetics of cytotoxicity to uninfected cells by propidium iodide staining and flow cytometry. The *agr*A mutant did not demonstrate significant detectable host cell death over the course of infection, and *sae*R, *aus*A and *aus*B mutants were significantly diminished in their ability to kill host cells. The *aus*B deficiency was readily complemented *in trans* (Fig 3a). Therefore, the absence of aureusimines resulted in drastically attenuated cytotoxicity of intracellular *S*. *aureus*. Since culture supernatants of wild-type and mutant bacteria induced similar levels of cell death (S5 Fig) and hemolysis was comparable (S7 Fig), we concluded that phevalin-dependent effects on epithelial cell death originated from intracellular bacteria.

During bacterial infections, neutrophils are usually the first cells recruited to the site of infection where they get activated and ingest bacteria. Since *S*. *aureus* has evolved mechanisms to evade neutrophils[35], we tested neutrophil killing by the pathogen. At 4 hours p.i. PMN cell death was significantly decreased in the *aus* mutants when compared to either wild type or the complemented *aus*B strain (Fig 3b). Similarly, primary human (Fig 3c and S5b Fig) and murine (S5c and S5d Fig) macrophages were killed in an NRPS-dependent manner even at low multiplicities of infection (MOI) of 5.

Bacterial replication in neutrophils was not affected by insertional inactivation within the *aus* operon, since CFUs increased between 1 and 4 hours after infecting PMN regardless of the strain background (S8 Fig). By contrast, *aus*A and *aus*B mutants were as susceptible to killing by macrophages as the *agr* mutant: recovered CFU counts of mutants in *aus*A or *aus*B diminished over the course of co-incubation with macrophages, whereas CFUs of wild-type (WT), complemented *aus*B mutant (*aus*B compl), and the *aus*A mutant supplemented with 10 μM phevalin (*aus*A+Phe) demonstrated increasing CFU values over the course of the experiment. By contrast, phevalin supplementation of the *agr*A mutant did not lead to increased CFU recovery (Fig 3d), thereby supporting the additional requirement of bacterial toxins for intracellular survival.

**Fig 4:**
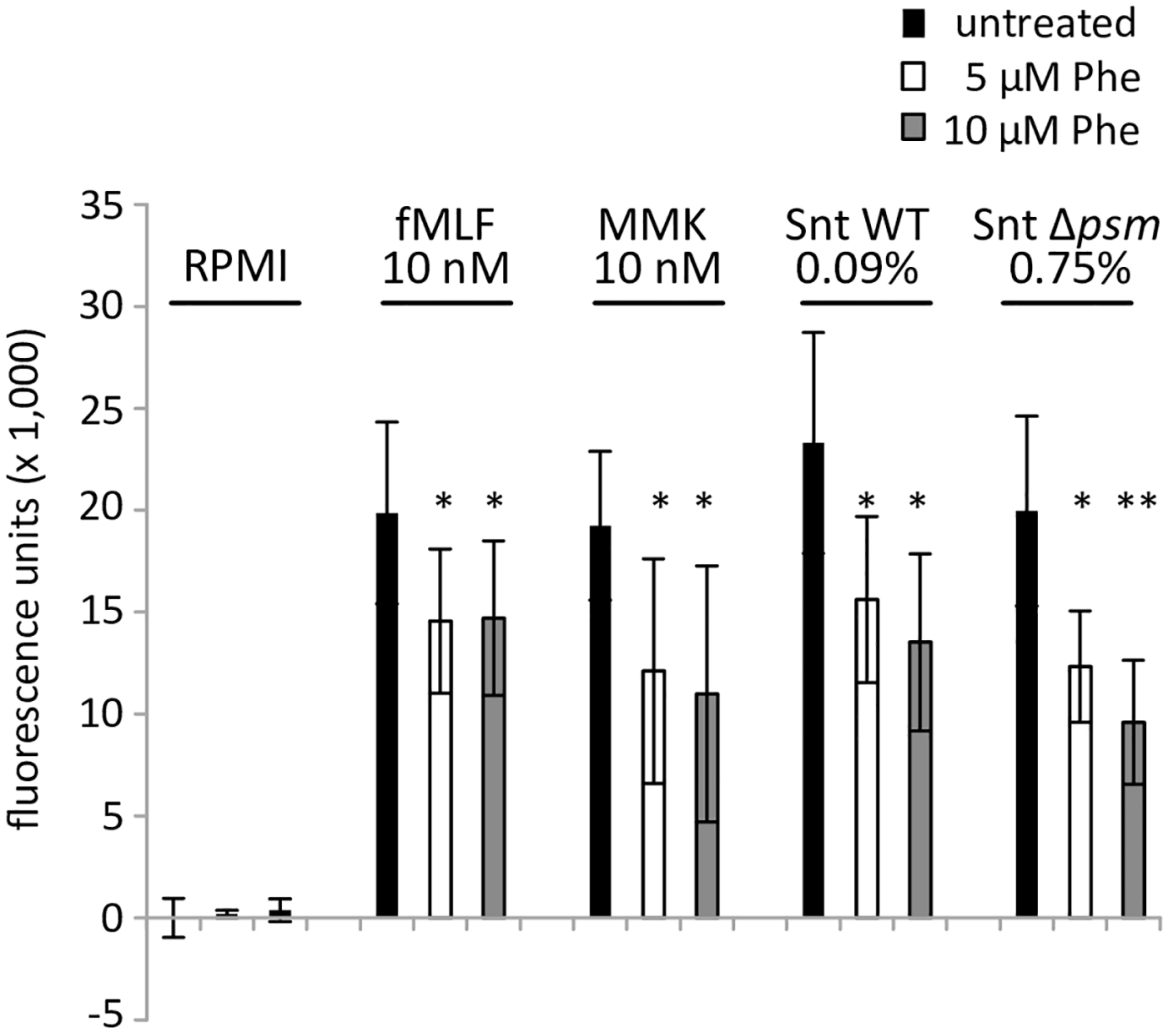
Phevalin inhibits activation of primary human neutrophils. Addition of synthetic phevalin inhibits activation of primary human neutrophils. By loading PMN with the calcium-sensitive fluorophore Fluo3 and flow cytometry we determined calcium flux upon addition of external stimuli as a proxy for PMN activation. Pre-incubation of PMN with 5 or 10 μM phevalin led to a significant decrease of calcium flux when PMN were stimulated with fMLF, MMK, or dilutions of culture supernatant of *S*. *aureus* wild type (0.09%) or a mutant in PSMs (USA300Δαβδ; 0.75%). Depicted is Fluo3 fluorescence units with RPMI-treated PMN set to 0.

As bacterial replication in neutrophils was not affected by *aus*AB mutations, we next investigated the effects of synthetic phevalin on activation of primary human neutrophils and assayed by flow cytometry the calcium flux, a hallmark of PMN activation. Interestingly, pre-incubation of PMN with phevalin led to a significant decrease of calcium flux when the neutrophils were challenged with formyl peptide receptor (FPR) 1 agonist fMLF, the FPR2 agonist MMK, or bacterial supernatants irrespective of the presence of phenol-soluble modulins (Fig 4). Formyl peptide receptors 1 and 2 are G-protein-coupled receptors on a variety of cells such as innate immune cells. Triggering of either FPR can activate the cells. Since phevalin addition inhibits neutrophil activation regardless of the addition of either specific agonist (MMK1 and fMLP, respectively) and since phevalin is active on the host cell level in epithelial cells which do not express FPRs, we reasoned that the inhibition of neutrophil activation might take place on the level of the downstream signaling cascade.

**Fig 5:**
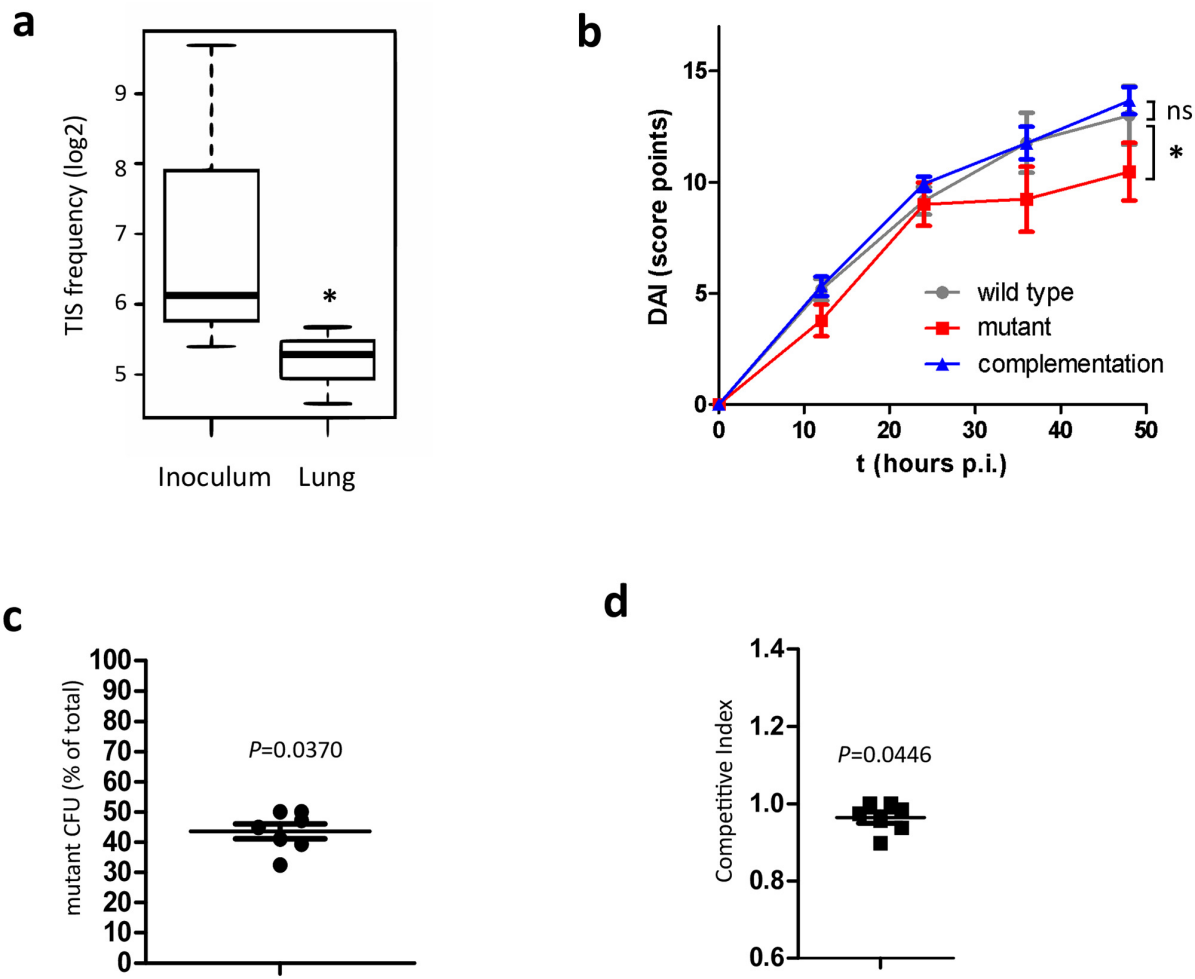
The *S*. *aureus* NRPS is required for full virulence in *S*. *aureus*-induced pneumonia. **a**) Next-generation sequencing of transposon insertion sites was used to detect changes of read frequencies in bacteria from transposon mutant recovered from mouse lungs when compared to the inoculum library. The box and whisker plot represents the median and range of the relative difference (log_2_ fold change) in frequency of transposon insertion sites detected in *aus*A (see also S5 Table). **b**) *aus*B mutants are attenuated in a pneumonia infection model. *S*. *aureus* JE2, an isogenic *aus*B mutant, as well as the *aus*B complementation strain were instilled intranasally into Balb/c mice (n = 10) and animal health (disease activity index, DAI) was continuously recorded over 48 hours at the indicated time points. The graph shows mean DAI ± SEM. Statistics were determined by linear models of DAI as a function of infection group and time; *P<0.05. **c**) In vivo competition of a 1:1 mixture of wild-type and the *aus*B mutant strain at a sublethal dosage shows that the mutant is outcompeted by the wild-type. Although these effects were subtle, *aus*B mutants are recovered at reduced frequencies (43.55% of total CFU, *P* = 0.037 for difference from 50%). **d**) The resulting competitive index of 0.96 is only slightly, but significantly different from 1 (*P* = 0.0446).

Further support for the importance of *aus*AB in virulence of *S*. *aureus* was provided by a transposon insertion site (TIS) sequencing screen in an animal model of pneumonia. We infected mice intranasally with a pool of transposon mutants of *S*. *aureus* 6850[36] and recovered bacteria from animal lungs one day after infection (S9a Fig). A transposon within *aus*A (insertion site at nucleotide 149,611; GenBank Accession NC_007793) demonstrated a 2^−2.7^ fold decrease of read frequencies in the recovered fraction when compared to the inoculum (per site analysis: *P* = 0.00025, adj. *P* = 0.024; per gene analysis 2^−2.15^ fold change; adjusted *P* = 0.093; S5 Table; Fig 5a).

Mutants within *gua*A, *pur*R, or *pur*M, other genes that had been previously shown to be essential for *S*. *aureus* survival in mouse models of infection, were also underrepresented in the lung samples (S5 Table). Upon recovery of the output library we compared its complexity and composition of that of the input, which were found to be highly correlated (adjusted R^2^ = 0.92, S9b Fig). In all inocula, we consistently recovered insertion sites from 1640 genes out of 2559 genes annotated for *S*. *aureus* 6850 [37]. The 919 discrepant genes might include essential genes and some lost due to minor technical bottlenecks arising from dilution of inoculum during infection (S9c Fig). We therefore excluded a major bottleneck effect to account for the depletion as well as growth defects of *aus* mutants to account for the differential recovery of mutant and wild type bacteria. Taking into account the 1640 consistently recovered gene mutants, we analyzed the differences in reads from individual mutants between the inoculum library and the recovered bacteria. Of these 123 were decreased in transposon insertion site (TIS) read abundance including *aus*A (S9d Fig and S5 Table). Moreover, within this list we found the non-coding RNA SSR42, a recently identified virulence regulatory RNA which was shown to be important for toxin production and virulence [36,38].

Since we excluded growth defects of *aus* mutants to account for the differential recovery of mutant and wild type bacteria (S9e Fig), our data suggested that the *aus* operon plays a distinct role in *S*. *aureus*-induced pneumonia and is required for efficient survival of the bacteria in mouse lungs.

We hence intranasally instilled mice with wild-type bacteria, *aus*B mutants as well as the *aus*B complementation strain and recorded disease activity index (DAI; S6 Table) over 48 hours of infection (Fig 5b). We observed a linear increase in disease severity over the first 24 hours post infection for all investigated strains. At the 24 hour time point, mice infected with the *aus*B mutant started to recover, which is supported by significant changes in DAI time course (*P* = 0.0213) as was established by a linear statistical model. After 48 hours, the mice were sacrificed and bacterial CFU from the tissue were determined by plating dilutions of tissue lysate (S10 Fig). Interestingly, the three strains were recovered at similar CFUs. We therefore performed a competition experiment during which mice were infected with a 1:1 mixture of the wild-type strain as well as the insertional *aus*B mutant. Wild-type *S*. *aureus* outcompeted the mutants as was indicated by a slightly, but significantly reduced retrieval of mutants when compared to the total inoculum (43.55% of total CFU, *P* = 0.037; Fig 5c) and therefore a competitive index (CI) that differed significantly, if slightly, from 1 (CI = 0.96; *P* = 0.0446; Fig 5d). These minute changes illustrate that the NRPS AusAB and its dipeptide product phevalin are possibly not major virulence factors of *S*. *aureus* during lung infection and thus support previous findings [21]. However, our results illustrate that the production of aureusimines may tip the balance in the tug-of-war between the pathogen and cellular host defenses in some cases (Fig 5b): for instance, we demonstrate that in epithelial cell lines, phevalin is involved in phagosomal escape of the pathogen. The phevalin dependency of cell death in macrophages and neutrophils suggests an additional function of the dipeptide in these immune cells and we are currently actively investigating the potential mode of action of phevalin and if in these cells phagosomal escape and host cell death are linked[12]. Since recent studies suggests that phagocytes are killed by *S*. *aureus* without prior translocation to the host cell cytoplasm [16,17] and not all cell types succumb to intracellular *S*. *aureus* [39] multiple, potentially cell- or tissue-specific intracellular survival strategies of *S*. *aureus* and molecular mechanisms of phevalin activity may exist.

Previously, the contribution of aureusimines to *S*. *aureus*-induced disease was unclear: the initial phenotypes observed by Wyatt et al.[20] were attributable to a secondary site mutation within the virulence regulator SaeRS[21]. In an isogenic background, however, evidence of virulence in infection models was lacking[21]. Both studies used *S*. *aureus* Newman, a strain lacking covalent cell wall anchorage of the main adhesins, Fibronectin-binding proteins A and B. *S*. *aureus* Newman thus hardly invades epithelial cells[40]. However, our data show that intracellularity of *S*. *aureus* is required to cause observable differences in phevalin-dependent phagosomal escape and subsequent epithelial cell death, whereas culture supernatants of wild type and mutant bacteria do not show profound differences in cytotoxicity. This also explains the importance of AusAB in the pneumonia model, in which the pathogen primarily encounters phagocytic cells such as alveolar macrophages and infiltrating neutrophils[41,42]. Altered pathogenesis of *aus* mutants in the animal lung is not caused by α-toxin, a main virulence factor in *S*. *aureus*-induced pneumonia[43], since it is not differentially regulated in *aus*-deficient mutants[20]. Further, α-toxin is not involved in phagosomal escape of host cells[44] (S4 Table).

Interestingly, mere addition of synthetic phevalin inhibited neutrophil activation independent of FPR receptors, thereby suggesting a host target of the pyrazinone. During phagosomal escape phevalin targets a host cellular function which is independent of calpain, although phevalin was first described to comprise a calpain inhibitor[25]. Host targets of phevalin are further supported by a study in which transcriptional changes upon phevalin treatment were observed in human keratinocytes[26].

*S*. *aureus* uses several small molecule products for virulence: the carotenoid staphyloxanthin is used to scavenge reactive-oxygen species, siderophores are important for iron acquisition and an autoinducing peptide pheromone is instrumental in quorum sensing. Aureusimines, cyclic dipeptides formed by a non-ribosomal multi-domain assembly line[45], are small molecule virulence modulators. Whereas such peptides often act as antibiotics and in interspecies competition, our data suggests that *S*. *aureus* exploits non-ribosomal dipeptides for an inter-kingdom modulation of host-pathogen interactions in order to survive within the mammalian host.

## Methods

### Bacterial culture conditions

*Escherichia coli* was grown in Luria-Bertani broth (LB; Oxoid). *Staphylococcus aureus* strains were routinely grown on Trypticase soy agar (TSA) or in Trypticase soy broth (TSB) supplemented with glucose (Oxoid). Media were supplemented with antibiotics where appropriate. For assessment of hemolysis *S*. *aureus* was grown on Columbia blood agar base (Oxoid) supplemented with 5% defibrinated sheep blood (Fiebig Nährstofftechnik, Germany).

Bacterial growth curves were determined with a TECAN infinite Pro 200 plate reader. Triplicates of each culture were used to inoculate 400 μL TSB to OD_600_ 0.1 and were grown up to 20 hours in a 48 micro well plate at 37°C with shaking at 180 rpm. Absorbance at 600 nm was recorded in 10 minute intervals.

### Aureusimine complementation

The plasmid p0182 was generated for genetic complementation of the insertional mutant in *aus*B, NE964. Briefly, the constitutively active promoter SarAP1 was amplified from *S*. *aureus* gDNA by the oligonucleotides SarAP1-F and SarAP1-R. The PCR product was TA-cloned and sequence verified. The SalI/KpnI Fragment with the promoter was generated by a restriction digest and inserted into accordingly opened pmRFPmars [46] resulting in pSarAP1-mRFP. The open reading frame of *ausB* (*S*. *aureus* USA300_FPR3757; GenBank Accession number NC_07793; Locus ID SAUSA300_00182) was amplified from genomic DNA of *S*. *aureus* JE2 using the oligonucleotides 0182_for and 0182_rev (S6 Table). The 672 kb insert was cloned in pCR2.1 TOPO TA vector (Invitrogen), transformed and propagated in *E*. *coli* DH5α. The sequence of *aus*B was verified by Sanger sequencing (SeqLab, Göttingen). A 669 bp DNA fragment was prepared by restriction and the purified fragment was ligated in the accordingly treated vector pSarAP1-mRFP. The vector was amplified in *E*. *coli* DH5α and subsequently was electroporated[47] into *S*. *aureus* RN4220[48], a strain that readily accepts and methylates foreign DNA thereby allowing to bypass the restriction barrier of wild-type *S*. *aureus*. From *S*. *aureus* RN4220 the methylated plasmid DNA was re-isolated and electroporated into the target strain, *S*. *aureus* NE964 (S4 Table). Selection for recombinant bacteria was performed by plating the cultures on TSA containing 10 μg/ml chloramphenicol.

Synthetic aureusimine B (Phevalin; CAS 170713-71-0) was obtained from SantaCruz Biotechnology (Heidelberg, Germany; sc-362711), dissolved in DMSO and stored in aliquots at -20°C. Titration of the biologically active phevalin concentration has to be performed for each new cell type.

### Phagosomal escape assays

Phagosomal escape was examined as described previously with modifications[9,12] that allowed automation of microscopy. Briefly, HeLa cells (HeLa 229, ATCC CCL-2.1) stably expressing YFP-CWT escape reporter construct[12,27] were grown in 24 well plates (ibidi μ-clear; #82406) in RPMI1640 medium (Invitrogen) supplemented with 10% FCS and 1 mM sodium pyruvate. The upper airway epithelial cell line S9 [49] was transduced with lentiviral particles stably integrating the escape marker YFP-CWT [12] and was cultivated in DMEM:F12 nut-mix supplemented with 10% FCS and penicillin/streptomycin (100 U/ml and 100 μg/ml, respectively). *S*. *aureus* cultures were grown overnight in TSB (37°C, 200 rpm). Cultures were diluted to an OD_600_ of 0.4 in 10 ml fresh TSB medium and incubated for another 1h at 37°C. Bacteria were harvested by centrifugation, labelled with 50 μg/ml TRITC (mixed isomers; MoBiTec) for 30 min at 37°C and were thoroughly washed to remove unbound dye. Bacteria were enumerated and used to infect HeLa at a multiplicity of infection (MOI) of 10. After a one hour co-cultivation, extracellular bacteria were removed by a 30 min treatment (37°C) with medium supplemented with 20 μg/ml lysostaphin (AMBI, Lawrence, NY, USA) and 100 μg/ml gentamicin (Invitrogen). Lysostaphin/gentamicin medium was aspirated and replaced with tissue culture medium containing 100 μg/ml gentamicin. After additional incubation (as indicated in text and figures) the cells were washed with 1x PBS and subsequently fixed with 4% paraformaldehyde for 1 hour at room temperature or overnight at 4°C.

Next, the samples were recorded with an Operetta Fluorescence Microscope (Perkin Elmer). For each well 10 non-overlapping images (each at 1360x1024 px; 675.3928 μm x 508.5311 μm) were acquired with a 20 x PLAN long working distance objective (NA 0.45). TRITC fluorescence (*S*. *aureus*) was imaged with the filter set "StdOrange1/Cy3" filter set (excitation: 520–550 nm, emission: 560–630 nm; 0.5 sec exposure). Fluorescence of the escape reporter YFP-CWT was recorded with the “SpBlue1/YFP" filter set (excitation: 490–510 nm, emission: 520–560 nm; 0.75 s exposure). Image analysis was performed with the built-in software “Harmony” (Perkin Elmer). Host cell cytoplasm and nuclei counts were identified by using the faint cytoplasmic fluorescence of YFP-CWT, which accumulates slightly within the nucleus of host cells. Within the area of host cell cytoplasm, bacteria as well as escape signals were detected and enumerated with “Find spots” in each of the recorded channels (Method “A”, relative spot intensity 0.1, Splitting coefficient 1). The mean relative escape scores were represented as YFP/CY3 ratios. In all assays, the escape-proficient *S*. *aureus* JE2 and its escape-deficient isogenic *agrA* mutant served as positive and negative controls, respectively. Statistical significance was calculated by Student’s t-test with wild type as the reference.

### UPLC detection of aureusimines

Bacteria culture samples were prepared after a previously established protocol[26]: 25 ml of TSB overnight culture of *S*. *aureus* was centrifuged at 4,000 rpm for 10 min at 4°C. Supernatant was collected and sterilized by passage through a 0.22 μm syringe filter. In glass tubes 2 ml of filtrate were mixed with an equal volume of 100% chloroform by vortexing. Samples were centrifuged at 3,000 g for 10 min at 4°C and the organic phase was transferred to a fresh glass tube. Solvent was evaporated in an extractor hood by a continuous flow through of pressurized air. The dried samples were resuspended in 100 μl of 20% DMSO.

For detection of aureusimines from epithelial cells, HeLa cells were incubated either in presence of 20 μM phevalin or were infected with bacteria (see above). At the time point of measurement cells were detached using trypsin/EDTA, 1.5 ml H_2_O was added and the sample was transferred to a glass reaction tube. Aureusimines were extracted as outlined above.

Samples were analyzed by LC-MS/MS using a Waters Acquity ultra-high-performance liquid chromatography system coupled to a Waters Micromass Quattro Premier triple quadrupole mass spectrometer (Milford, MA, USA) equipped with an electrospray interface (ESI).

Aureusimines were separated by reversed-phase chromatography using an Acquity BEH C18 column (50 x 2.1 mm, 1.7 μm particle size with a 5 x 2.1 mm guard column; Waters; Milford, MA, USA) and a solvent system consisting of water containing 0.1% formic acid (solvent A) and acetonitrile (solvent B). The injection volume was 5 μL per sample. A gradient elution was performed at a flow rate of 0.25 mL min–1 starting from 1% to 100% solvent B within 5 min and a column temperature of 40°C.

Aureusimines were detected by multiple reaction monitoring (MRM), instrument parameters for ionization and collision induced dissociation (CID) were optimized by flow injection of phevalin. The electrospray source was operated in the positive electrospray mode (ESI+) at a temperature of 120°C and a capillary voltage of 2.75 kV. The cone voltage (CV) was adjusted to 40 V and nitrogen was used as desolvation and cone gas with flow rates of 800 L h–1 at 400°C and 10 L h–1, respectively. Fragmentation was carried out using argon as collision gas at a pressure of approximately 3 x 10–3 bar and a collision energy (CE) of 22 eV. For each compound, three specific fragments were monitored (phevalin: m/z 229 > 214, m/z 229 > 159, m/z 229 > 81; tyrvalin: m/z 245 > 230, m/z 245 > 175, m/z 245 > 81) with a dwell time of 25 ms per MRM transition.

### Epithelial cell death assays

HeLa 229 (CCL-2.1) were obtained from ATCC. Cells were grown on 12 well plates in RPMI1640 medium (Invitrogen) supplemented with 10% FCS (PAA), 1 mM sodium pyruvate (Invitrogen). *S*. *aureus* cultures were grown overnight in TSB and the cultures were diluted to an OD_600_ of 0.4 in 10 ml fresh TSB medium and incubated for 1 h at 37°C (200 rpm). Bacteria were harvested by centrifugation, washed with PBS, resuspended in tissue culture medium and used for infection at a MOI of 10. Extracellular bacteria were removed by lysostaphin/gentamicin treatment 1 hour post infection as outlined above and were further incubated for 3 hours. At 4 hours post infection, supernatants for each well were collected, cells were detached from the substratum using TrypLE Express (Invitrogen), and trypsinization was stopped by re-addition of the previously collected culture supernatants. Cell suspensions were transferred to reaction tubes and cells were collected by centrifugation at 500 x g for 5 min. The cells were gently resuspended in FACS labelling solution (10 mM HEPES pH 7.4, 140 mM NaCl, 5 mM CaCl_2_, and 1% [v/v] propidium iodide). The reaction was incubated in the dark for 10 minutes at room temperature, cells were diluted 1:5 with labelling buffer, and analyzed immediately using an Accuri C6 flow cytometer (BD). The wild type and non-infected HeLa cells were used as references to compare the cytotoxicity. The mean percentages of PI-positive events were plotted and statistical analysis was performed by fitting the values into a linear model, where the responses were time and infection groups. ANOVA and Tukey’s post hoc analysis were performed to assess individual differences.

### *S*. *aureus* neutrophil cytotoxicity and bacterial killing assays

Primary human polymorphonuclear leukocytes (PMNs) were isolated from venous blood of healthy adult volunteers as described[50], were harvested by centrifugation at 1,000 rpm for 5 minutes and resuspended in 1x Hank’s Balanced Salt Solution (HBSS). Neutrophil killing by intracellular *S*. *aureus* was determined as described[51]. At indicated times post-infection neutrophils were pelleted by centrifugation. Supernatants were collected and lactate dehydrogenase (LDH) was measured using the Cytotoxicity Detection Kit PLUS (Roche).

To examine killing of *S*. *aureus* by neutrophils, PMN were lysed with sterile water (pH 11.0) for 5 minutes. Serial dilutions of the bacterial suspension were plated on TSA and bacterial colonies were counted after overnight incubation at 37°C.

### Determination of neutrophil activation by detection of calcium flux

Calcium fluxes in PMN were analyzed by loading the cells with Fluo-3-AM (Molecular Probes) and monitoring fluorescence with a FACScalibur flow cytometer (Becton Dickinson) as described[52]. The synthetic chemoattractants fMLF and MMK were used at final concentrations of 10 nM to stimulate calcium fluxes in PMN, while *S*. *aureus* culture supernatants of staphylococcal strains LAC [53] and the PSM-deficient mutant LAC Δαβδ [54] were used in the indicated concentrations. In order to determine a potential effect of the aureusimine phevalin on PMN calcium flux, cells were pre-incubated with concentrations of 5 or 10 μM for 30 min at RT prior to the experiments. Calcium flux of buffer control corrected samples was expressed as relative fluorescence units (RFU) from measurements of 2,000 events.

### *S*. *aureus* macrophage killing and cytotoxicity assay

Isolation and differentiation of primary human macrophages was performed as described before[55]. In brief, Peripheral blood mononuclear cells (PBMCs) were collected following centrifugation of whole blood collected from healthy individuals over a Ficoll-Paque (GE Healthcare) gradient at 200 x g for 30 min and washing with PBS containing 1 mM EDTA. Centrifugation at each step was performed for 10 min at 300 x g without brakes. PBMCs were resuspended in RPMI medium supplemented with 250 ng/ml phytohemagglutinin (Sigma Aldrich), and incubated at 37°C and 5% CO_2_ for 24 to 36 hours. Monocytes were allowed to differentiate into macrophages for 6 days, in medium supplemented with 50 ng/ml MCS-F (Affymetrix) for the first 3 days followed by 100 ng/ml. Macrophages were infected with *S*. *aureus* JE2 wild type, *aus*A, *aus*B, *agr*A mutants as well as an *aus*B complemented strain at an MOI of 5, to determine bacterial survival when challenged with macrophages. At each time point cells were washed once with PBS and lysed using alkaline water, pH 11 for 5 min at room temperature to release ingested bacteria. Dilution series of the complete cell lysate were plated on TSA to enumerate bacterial numbers. When cytotoxicity of internalized bacteria strains in macrophages was to be determined, macrophages were infected with an MOI of 20. Following centrifugation at 1000 rpm for 10 min, phagocytosis was allowed for 30 min at 37°C and 5% CO_2_. Medium was then exchanged for medium supplemented with 20 μg/ml Lysostaphin for 30 min to eradicate any residual extracellular bacteria. Medium was exchanged again for medium without antibiotics and cells were incubated at 37°C and 5% CO_2_ for another 60 min. At that time point 2 x 100 μl of medium were taken off to determine levels of lactate dehydrogenase (LDH) using the Cytotoxicity Detection Kit PLUS (Roche).

### Preparation of *S*. *aureus* for pulmonary infections

Desired *S*. *aureus* strains were revived from frozen stocks by plating on TSA with appropriate antibiotics whenever required. Colonies were picked and grown in TSB overnight at 37°C with shaking at 180 rpm in air. The overnight culture was transferred into a flask containing 50 ml TSB (OD_600_ 0.05) and incubated for 3.5 hours at 37°C with shaking at 180 rpm in air. The bacteria were harvested by centrifugation at 4°C and resuspended in 20 ml TSB containing 15% glycerol. The bacterial suspension was divided into 2 ml aliquots and stored at -80°C until use. The bacterial stocks were quantified for CFUs and also titrated for lethal and sub-lethal dosage for infection in mice. For infections bacteria from glycerol stocks were thawed, transferred to pre-warmed 50 ml TSB media and incubated at 37°C for 30 minutes. The bacteria were washed twice with 1x PBS and resuspended in 1 ml PBS. The optical density at 600 nm was determined and bacterial numbers were assessed by way of comparison with reference growth curves that were established for each strain. Bacteria were diluted to contain the desired number of CFU in 20 μl of PBS and aliquots were plated on TSB agar to confirm CFUs.

### Pneumonia mouse infection model

Female Balb/c mice aged 6 weeks were purchased from Janvier Labs, (Saint-Berthevin, France) and were kept in individually ventilated cages on a normal diet in six groups of 5. At 8 weeks, groups of 10 mice were infected intranasally with a bacterial suspension containing 2 x 10^8^ CFU in 20 μl PBS with either *S*. *aureus* JE2, an isogenic *aus*B mutant or the *aus*B complementation strain in a pneumonia model of infection.

After infection, mice were monitored for weight loss and signs of infection or severe disease every 12 hours. A disease activity index (DAI; see S6 Table) for each individual mouse was defined based on these observations and was plotted (Fig 5b). We observed collinearity between disease severity and time for wild-type bacteria, which was confirmed by a linear model in R (r^2^ = 0.7016; F(4,60) = 35.27, *P* = 3.864e-15). When analyzing all DAI scores as a function of infection group and time, the model was significant (F(6,188) = 61.14, *P* < 2.2e-16).

For enumeration of CFU load in lungs, complete lungs were removed 48 hours after start of infection, homogenized in 1X PBS (GentleMACS, M-tubes, Miltenyi Biotec). The lung lysates were serially diluted, plated on TSA and incubated for 24 hours at 37°C. Bacterial colonies were counted and bacterial load per lung was determined. Statistical analysis was performed using one-way ANOVA.

### Murine bone marrow-derived macrophage (mBMDM) cytotoxicity

mBMDMs were cultivated as previously described [56]. Briefly, hind limbs from 6–8 week old C57BL/6 donor mice were isolated followed by separation of femur and tibia. The ends of the bones were clipped and bone marrow was flushed out with BMM (RPMI 1640 containing 25 mM HEPES, 10% FCS, 1% penicillin-streptomycin, 1X sodium pyruvate). Bone marrow preparations from donor mice were individually processed. The obtained cells were mixed thoroughly and passed through a 70 μm strainer. Cells were washed with 1X Dulbecco’s PBS and resuspended in BMM medium containing 10% of L929 mouse fibroblast-conditioned medium. Cells were seeded at a density of 5 x 10^7^ cells per plate into sterile 15 cm petri dishes and were differentiated for seven days. BMM medium was exchanged every 2–3 days. On day 7, the cells were washed twice with 1X Dulbecco’s PBS and after addition of ice-cold 1X Dulbecco’s PBS the cells were kept at 4°C in order to detach the cells.

For infection with *S*. *aureus*, mBMDMs were seeded in 24-well plates at a density of 2 x 10^5^ per well in RPMI 1640 containing 25mM HEPES, 10% FCS, 1X sodium pyruvate to starve the cells for macrophage colony-stimulating factor (M-CSF).

On Day 8, mBMDMs were infected with *S*. *aureus* at the indicated MOI for 30 minutes, followed by addition of 20 μg/ml lysostaphin and a 30 minute incubation to eradicate extracellular bacteria. Macrophages ells were washed with 1X Dulbecco’s PBS and were further incubated with RPMI 1640 containing 25mM HEPES, supplemented with 1% FCS, 1X Sodium Pyruvate. At every time point, mBMDM cytotoxicity was analyzed by using Roche Cytotoxicity Detection Kit^PLUS^ (LDH), as per manufacturer’s instructions.

### In vivo competition experiment

Regarding in vivo competition experiment, female Balb/c mice (18–22 g, Charles River, Sulzfeld, Germany) were infected with a 1:1 mixture of *S*. *aureus* JE2 wt and the insertional *aus*B mutant. Lungs were harvested 48 hours after start of infection, homogenized and plated in serial dilutions on both TSB and TSB + Erythromycin agar plates. Since only the mutant strain harbors erythromycin resistance, we calculated the ratio of resistant (*aus*B) to sensitive (wild type) colonies. Only mice with at least 1000 CFU per lung (technical detection limit) were evaluated.

### *S*. *aureus* mutant library screen in mouse lung

Himar1 transposon mutant libraries in *S*. *aureus* 6850 [57] were generated as previously described[58] and kept as frozen glycerol stocks. For administering in mice, bacterial stocks were revived as outlined above. Appropriate dilutions containing the desired number of CFUs in 20 μl of PBS and aliquots were plated on TSB agar to confirm CFUs. The mutant library to be used was titrated for lethal and sub-lethal dosage in mice. For screening experiment, groups of 3 female BALB/c mice were administered intra-nasally with 2x10^8^ CFUs/ 20 μl and the same volume was plated on TSB agar to retrieve the input library. After 24 hours post administration, mice were euthanized and bacteria were recovered by plating the homogenized lungs (GentleMACS, M-tubes, Miltenyi Biotec, Germany) on TSB agar. Bacteria were harvested from the plates by scraping.

Transposon insertion site sequencing was performed as previously described [36]. Shortly, bacterial DNA was prepared from the inoculum library [36] or from bacteria recovered from the lungs of three mice, which served as three biological replicates. The DNA was fragmented, end-repaired and A-tailed. Thereafter, multiplexing adaptors consisting of the annealed oligonucleotides MultiPlex-Y-Adapt_f and MultiPlex-Y-Adapt_r were ligated to the DNA fragments. We next enriched for fragments containing *himar*1 transposon insertion sites by linear PCR with the primer TnSeq-HimarPCR, which binds to the *himar*1. In a second PCR we added oligonucleotides containing Illumina barcodes (MP-TnSeq_Index, S2 Table). The products were purified with AMPure beads and the barcoded libraries were sequenced on an Illumina Hi-Seq 2500 platform (single read, with index read) with the transposon-specific oligonucleotide Himar1-Seq. Illumina adapter sequences were removed via cutadapt version 1.2.1 [59] and were checked for the sequence pattern ‘CAACCTGT’ originating from the transposon end. Only reads containing that specific sequence with maximally one mismatch or gap and a minimum length of 16 nucleotides were used for further analyses. The remaining reads were mapped on the *Staphylococcus aureus* 6850 genome (GenBank accession CP006706) via Bowtie2 version 2.1.0[60].

### RNA extraction and real-time PCR

Bacterial mRNA of in vitro shaking cultures was extracted using TRIzol from bacteria grown in TSB for 2 (exponential phase) and 8 hours (stationary phase) after an inoculation to OD_600_ 0.1 from an overnight culture. After determination of RNA concentrations using a NanoDrop spectrophotometer (Nanodrop Technologies) DNA digestion (Turbo DNA-free Kit, Ambion) as well as reverse transcription (RevertAid First Strand cDNA Synthesis Kit, Thermo Scientific) were performed according to manufacturer’s guidelines. Real time PCR was performed on a StepOne Plus Real Time PCR system (Applied Biosystems) using SYBR Green PCR master mix. Primers used are listed in S2 Table.

### Statistical analyses

Normality of data distribution was checked by Shapiro Wilk’s test and wherever parametric or non-parametric tests for significances were applied.

For comparisons of phagosomal escape assays, CFUs from phagocytes, epithelial cells and mouse lung tissue, assays of neutrophil activation, cytotoxicity assays or real-time PCR results, significances were determined by Student’s t-test. For time-dependent epithelial cytotoxicity assays and DAI analysis of mice, we used linear models and ANOVA with time and infection group as factors. Tukey’s post-hoc analysis was performed for deducing individual differences. For macrophage killing assays, CFUs at t0, i.e. 15 minutes post-infection, were normalized to 100% and fold increase during the further time points were calculated. Wilcoxon rank sum tests were applied to comparison pairs for calculating significance. Mouse survival was assessed by fitting the observations into Kaplan Meier estimation curves.

For Tn-seq data analysis, the inoculum libraries were compared to the recovered mutant libraries obtained from the mouse lungs, in biological triplicates. Gene-wise analysis was done by comparing mean read abundances of TIS originating from each gene in input and output. Site-wise analysis was performed comparing the reads originating from individual TIS in input and output. For reduction of background noise, only sites with at least two independent detections have been statistically evaluated. Genes with very low mean normalized read depth (mnrd< 8) were excluded from analysis. Identification of depleted/enriched mutants was performed using a negative binomial regression model (implemented in DESeq2 version 1.6.2[61]). The *P*-values were corrected for multiple testing [62] and genes showing an adjusted *P*-value < 0.1 were reported as significantly affected. To exclude bottleneck effects or stochastic loss essential genes were assessed by comparing all the input libraries with each other and only the genes that appeared in all inputs were considered for further analyses. TIS read abundances obtained from each gene in the mutant library inoculum were compared to that of the recovered output from lungs. Both series were fitted into a simple linear model of regression and were found to be highly similar in complexity (R^2^ = 0.92, F = 2464 on 1 and 2206 Degrees of Freedom, *P* < 2.2 x 10^−16^). The differences obtained from the resultant analysis is represented by pie charts, displaying the TIS from each gene being enriched, depleted or unchanged in the Tn-seq screen.

### Ethics statements

Biosecurity (permission number AZ 50–8791.30.30) as well as the animal studies and protocols (permission number AZ 2532-2-155) were approved by the local government of Lower Franconia, Germany. Animal studies were performed in strict accordance with the guidelines for animal care and experimentation of the German Animal Protection Law and with EU directive 2010/63/EU. Experiments using human blood were approved by the Ethics Committee of the University of Würzburg (AZ 2015091401). Blood was drawn from healthy adult volunteers, who provided written informed consent.

## Supporting Information

S1 Fig *S*. *aureus* phagosomal escape assay by automated fluorescence microscopy. **a**) Phagosomal escape of red-fluorescent *S*. *aureus* (red) was microscopically detected by cells stably expressing the fluorescent reporter YFP-CWT (green) in the host cell cytoplasm. The cell wall targeting domain (CWT) of the metallopeptidase lysostaphin shows strong affinity for the bacterial cell wall and is efficiently recruited to *S*. *aureus* upon translocation to the cytoplasm. The arrow indicates a single, non-escaped *S*. *aureus*. **b**) Flowchart of data acquisition. Using an Operetta Fluorescence Microscope we recorded TRITC/Cy3 fluorescence (*S*. *aureus*, red) and YFP-CWT (phagosomal escape, green) for multiple fields per well. Phagosomal escape is evident by recruitment of YFP-CWT to *S*. *aureus*. Image analysis was performed with the built-in Harmony software, which identified host cell cytoplasms, nuclei, as well as spots in either green or red channel. The mean relative escape scores were represented as YFP/Cy3 ratios. **c)** AusAB-dependent escape in upper airway epithelial cells. Escape rates of mutants within *aus*A and *aus*B are reduced to levels of the negative control, *pmt*C. Complementation of *aus*B expression (*aus*B compl) restores the escape phenotype. Statistical analysis was performed by t-test. *P<0.05; ***P<0.001.
(TIF)

S2 Fig UPLC Detection of aureusimines in extracted culture supernatants of *S*. *aureus*. **a**) Production of aureusimine A (tyrvalin, retention time 3.36) and B (phevalin, retention time 4.06) is lost in the *S*. *aureus aus*A and *aus*B mutants, but is readily detected in wild type (WT) and complemented *aus*B mutant. An *agr*A mutant was capable of aureusimine production, although at slightly reduced amounts. **b**) After phevalin treatment, the pyrazinone is associated with mammalian cells even after extensive washing. Phevalin was added to culture medium of HeLa. After 60 min incubation, the supernatant was aspirated and the monolayer was profoundly rinsed with PBS. After extraction of the cells with chloroform, phevalin was detected by UPLC (bottom lane), whereas controls were negative for the molecule. **c**) At 4 hours p.i., phevalin of bacterial origin can be readily extracted from cells infected with wild type *S*. *aureus* (bottom lane), but is absent in uninfected cells, as well an cell infected with an *aus*A mutant. Aureusimines were detected by multiple reaction monitoring (MRM), instrument parameters for ionization and collision induced dissociation (CID) were optimized by flow injection of phevalin. All chromatograms depict intensities per cent (Y-axis) and retention time (X-Axis, min). Y-axes were fixed for the highest signal throughout the samples to allow for comparison of intensities. Chromatograms in (a,b) display the combination of three MRM measurements each for tyrvalin and phevalin, respectively. To improve sensitivity of detection of phevalin produced by intracellular *S*. *aureus* in (c), only the phevalin specific transition is displayed.
(TIF)

S3 Fig Phevalin does not affect the structural integrity of *S*. *aureus*-containing endosomes. Formaldehyde fixed bacteria were used to infect HeLa YFP-CWT escape reporter cells. Phevalin treatment did not lead to appearance of escape signals, indicating that treatment with the pyrazinones did not alter integrity of endosomal membranes. Bar graphs show the mean of three independent experiments ± SD. Statistical analysis was performed by t-test.
(TIF)

S4 Fig Phevalin does not affect invasiveness of *S*. *aureus* into HeLa cells. By automated microscopy we enumerated TRITC-labelled intracellular bacteria 3 hours post infection. Wild type *S*. *aureus* (WT), mutants within *aus*A or *aus*B, as well as the complemented *aus*B strain (*aus*B compl) did not show significant changes in intracellular bacteria, regardless of addition of external phevalin to culture medium one hour prior to infection. Bar graphs show the mean of three independent experiments ± SD. Statistical analysis was performed by t-test.
(TIF)

S5 Fig NRPS-dependent cytotoxicity is mediated by intracellular bacteria. **a)** Culture supernatants do not exhibit aureusimine-dependent differences in cytotoxicity. We collected culture supernatants of the bacteria and—in addition—of an *agr*A mutant by centrifugation and sterile filtration and incubated HeLa with a 1:10 dilution of supernatants in RPMI1640 containing 1% FCS. Cell death was measured by LDH release and is plotted as per cent of the positive control. Whereas supernatants of the *agr*A mutant was apathogenic, no significant changes were observed for the remainder of the strains. **b-d**) *S*. *aureus* cytotoxicity against primary macrophages. Host cell death rates were determined in hMDM 4 hours p.i. (**b**) and murine bone marrow-derived macrophages (mBMDM) 2 and 4 h p.i. (**c,d**). Cell death was measured by LDH release as percentage relative to the complete lysate (positive control) and supernatant of uninfected cells (set to 0% cell death, negative control. Graphs show the mean of at least three independent experiments ± SD. Statistical analyses were performed by t-test. *P < 0.05; **P<0.01; ***P < 0.001.
(TIF)

S6 Fig RT-PCR demonstrates enhanced transcription of the *aus*AB operon in the stationary phase of growth. RNA was prepared from *S*. *aureus* strain LAC, JE2 as well as the JE2 mutants in *aus*A or *agr*A in exponential and stationary growth phases. Transcript abundances of either *aus*A (left panel) or *aus*B (right panel) were determined by RT-PCR. Statistical analysis was performed by t-test. *P<0.05; **P<0.01; ***P<0.001.
(TIF)

S7 Fig Deficiency in *aus*A and *aus*B does not influence hemolysis. Hemolysis on Columbia blood agar is comparable between *S*. *aureus* wild type (WT) and insertional mutants of *aus*A and *aus*B, whereas an *agr*A mutant is non-hemolytic.
(TIF)

S8 Fig Phevalin does not influence intracellular CFU in neutrophils. Bacterial replication in neutrophils was determined by CFU recovery assays and was not affected by a mutation in the *aus* operon. Recovered CFU increased between 1 and 4 hours after infecting PMN with the bacteria regardless of the strain background.
(TIF)

S9 Fig Transposon mutant library screening. **a**) Balb/c mice were infected intranasally with a mariner transposon mutant library of *S*. *aureus* 6850 comprising ~25,000 mutants. Viable bacteria were recovered from murine lungs 24 h p.i. and pools of recovered bacteria as well as the respective inoculum were analyzed by transposon insertion site sequencing. Changes in transposon insertion sites frequencies were analyzed for mutants enriched or depleted in the pneumonia model. **b**) Correlation analysis of Tn-seq reads from all TIS depicts high similarity (R^2^ = 0.92) in composition and complexity between the inocula and mutant library recovered from mouse lungs. **c**) 1640 out of 2559 genes within *S*. *aureus* 6850 were consistently recovered in all inocula used. **d**) Gene-wise comparison of mutants recovered from mouse lungs with the inocula displays the number of genes that had either depleted, enriched, or unchanged TIS read abundances 24 hours post infection. **e)** *aus* mutants do not exhibit growth defects when compared to wild type *S*. *aureus*. Triplicates of each culture were used to inoculate 400 μL TSB to OD_600_ 0.1 and were grown up to 20 hours in a 48 micro well plate at 37°C with shaking at 180 rpm. Bacterial growth curves were determined with a TECAN infinite Pro 200 plate reader. Absorbance at 600 nm was recorded in 10 minute intervals.
(TIF)

S10 Fig In vivo effects of *aus*B inactivation. **a**) The insertional *aus*B mutant causes less severe disease in the pneumonia model as is indicated by reduced weight loss in mice infected with the mutant. Disease severity is restored upon complementation of *aus*B in trans (compl.). **b**) Bacterial CFUs were recovered from lung tissue of infected mice 48 hours p.i. by plating serial dilutions of lysate. Recovered CFUs are not significantly reduced in the mutant when compared to the wild type (wt) or the complemented mutant (compl.).
(TIF)

S1 Table Bacterial strains used in this study. (PDF)

S2 Table Plasmids used in the study. (PDF)

S3 Table PCR and Adaptor Oligonucleotides. (PDF)

S4 Table Escape efficiencies of tested *S*. *aureus* mutants. (PDF)

S5 Table *S*. *aureus* transposon mutants that were significantly depleted or enriched in a murine pneumonia model. (PDF)

S6 Table Clinical score determination of mice to assess severity of disease. (PDF)
