# Supplementary figures and images for: Staphylococcus aureus Exploits a Non-ribosomal Cyclic Dipeptide to Modulate Survival within Epithelial Cells and Phagocytes
Source: PLoS Pathog. 2016 Sep 15;12(9):e1005857. doi: 10.1371/journal.ppat.1005857 (PMC5025175; doi:10.1371/journal.ppat.1005857)

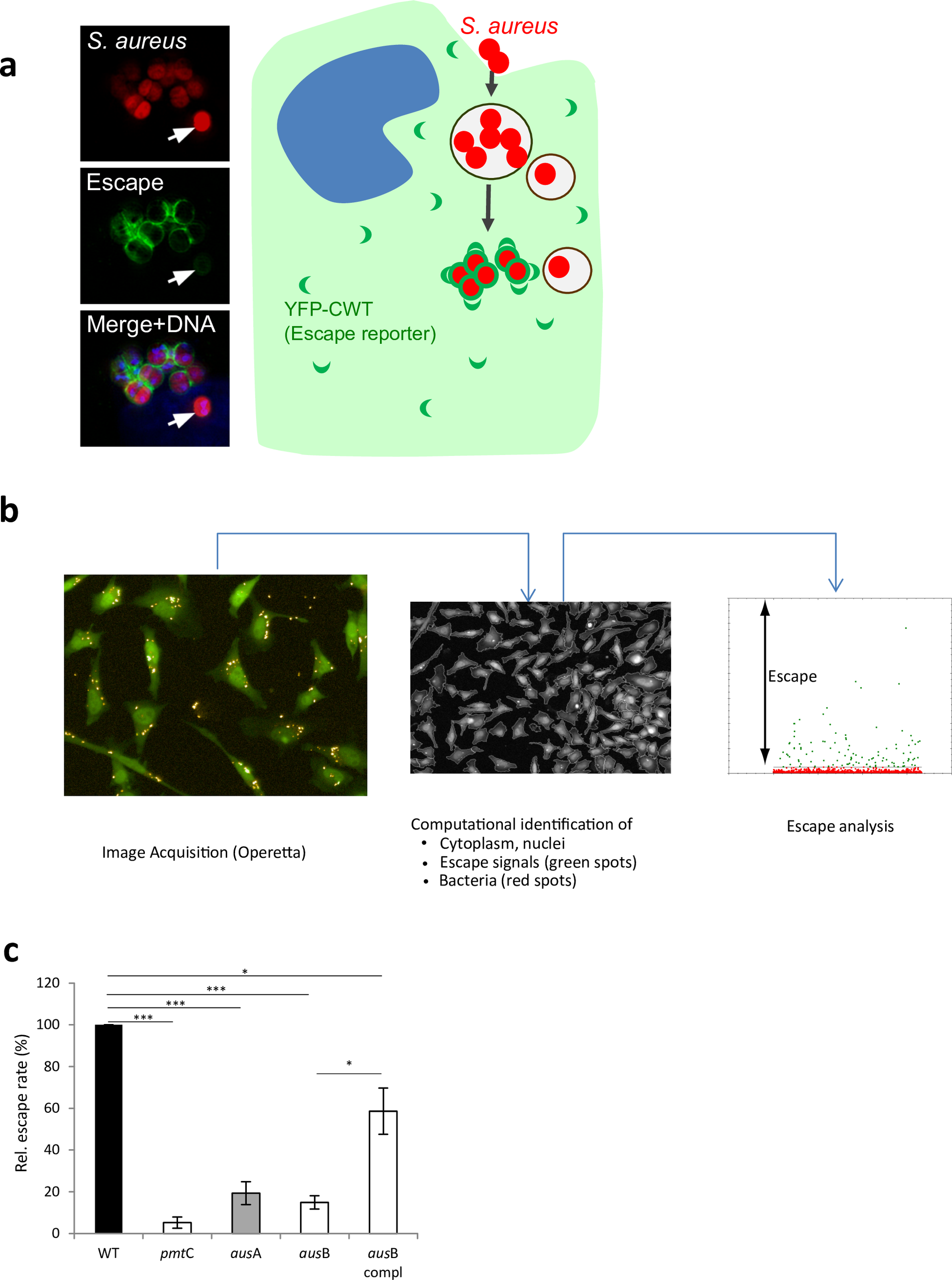

Supplement: S1 Fig — a) Phagosomal escape of red-fluorescent S. aureus (red) was microscopically detected by cells stably expressing the fluorescent reporter YFP-CWT (green) in the host cell cytoplasm. The cell wall targeting domain (CWT) of the metallopeptidase lysostaphin shows strong affinity for the bacterial cell wall and is efficiently recruited to S. aureus upon translocation to the cytoplasm. The arrow indicates a single, non-escaped S. aureus. b) Flowchart of data acquisition. Using an Operetta Fluorescence Microscope we recorded TRITC/Cy3 fluorescence (S. aureus, red) and YFP-CWT (phagosomal escape, green) for multiple fields per well. Phagosomal escape is evident by recruitment of YFP-CWT to S. aureus. Image analysis was performed with the built-in Harmony software, which identified host cell cytoplasms, nuclei, as well as spots in either green or red channel. The mean relative escape scores were represented as YFP/Cy3 ratios. c) AusAB-dependent escape in upper airway epithelial cells. Escape rates of mutants within ausA and ausB are reduced to levels of the negative control, pmtC. Complementation of ausB expression (ausB compl) restores the escape phenotype. Statistical analysis was performed by t-test. *P<0.05; ***P<0.001. (TIF) [file ppat.1005857.s001.tif]

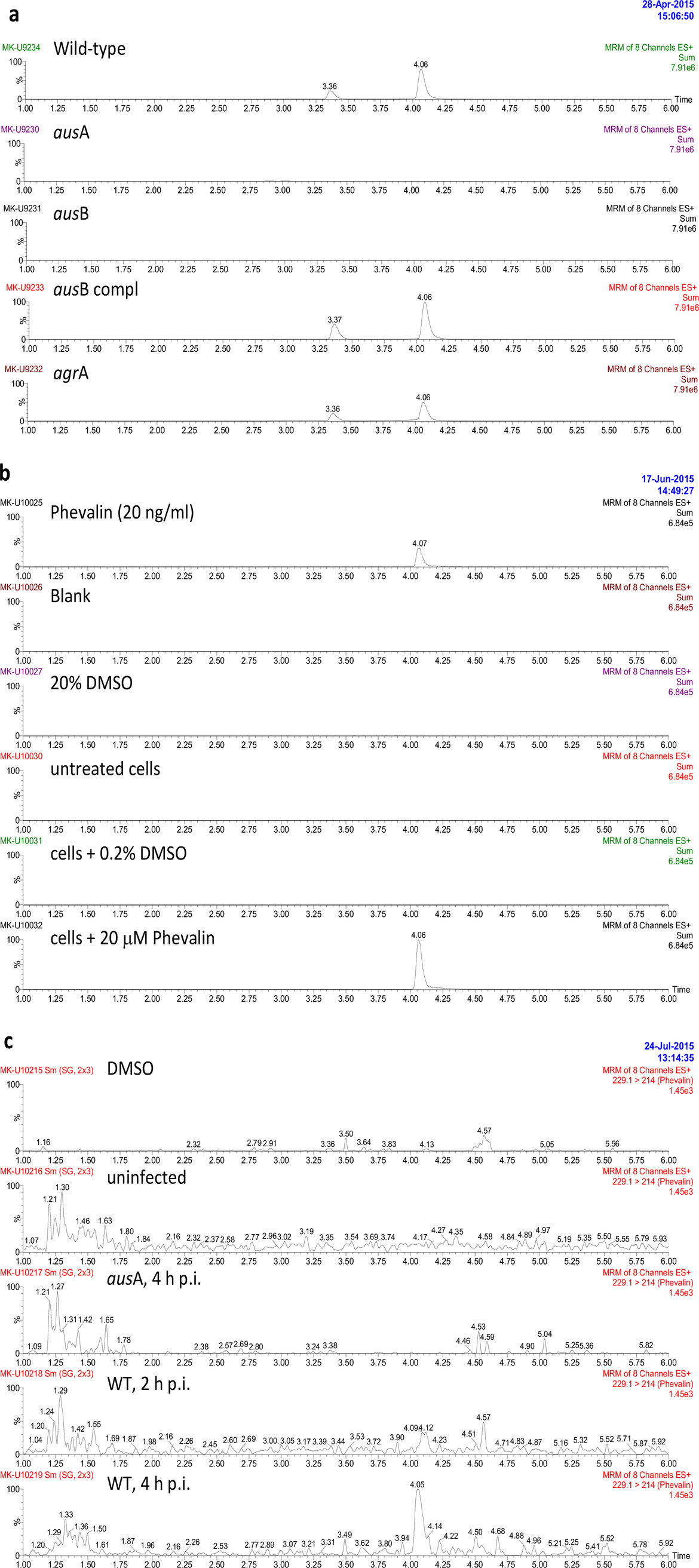

Supplement: S2 Fig — a) Production of aureusimine A (tyrvalin, retention time 3.36) and B (phevalin, retention time 4.06) is lost in the S. aureus ausA and ausB mutants, but is readily detected in wild type (WT) and complemented ausB mutant. An agrA mutant was capable of aureusimine production, although at slightly reduced amounts. b) After phevalin treatment, the pyrazinone is associated with mammalian cells even after extensive washing. Phevalin was added to culture medium of HeLa. After 60 min incubation, the supernatant was aspirated and the monolayer was profoundly rinsed with PBS. After extraction of the cells with chloroform, phevalin was detected by UPLC (bottom lane), whereas controls were negative for the molecule. c) At 4 hours p.i., phevalin of bacterial origin can be readily extracted from cells infected with wild type S. aureus (bottom lane), but is absent in uninfected cells, as well an cell infected with an ausA mutant. Aureusimines were detected by multiple reaction monitoring (MRM), instrument parameters for ionization and collision induced dissociation (CID) were optimized by flow injection of phevalin. All chromatograms depict intensities per cent (Y-axis) and retention time (X-Axis, min). Y-axes were fixed for the highest signal throughout the samples to allow for comparison of intensities. Chromatograms in (a,b) display the combination of three MRM measurements each for tyrvalin and phevalin, respectively. To improve sensitivity of detection of phevalin produced by intracellular S. aureus in (c), only the phevalin specific transition is displayed. (TIF) [file ppat.1005857.s002.tif]

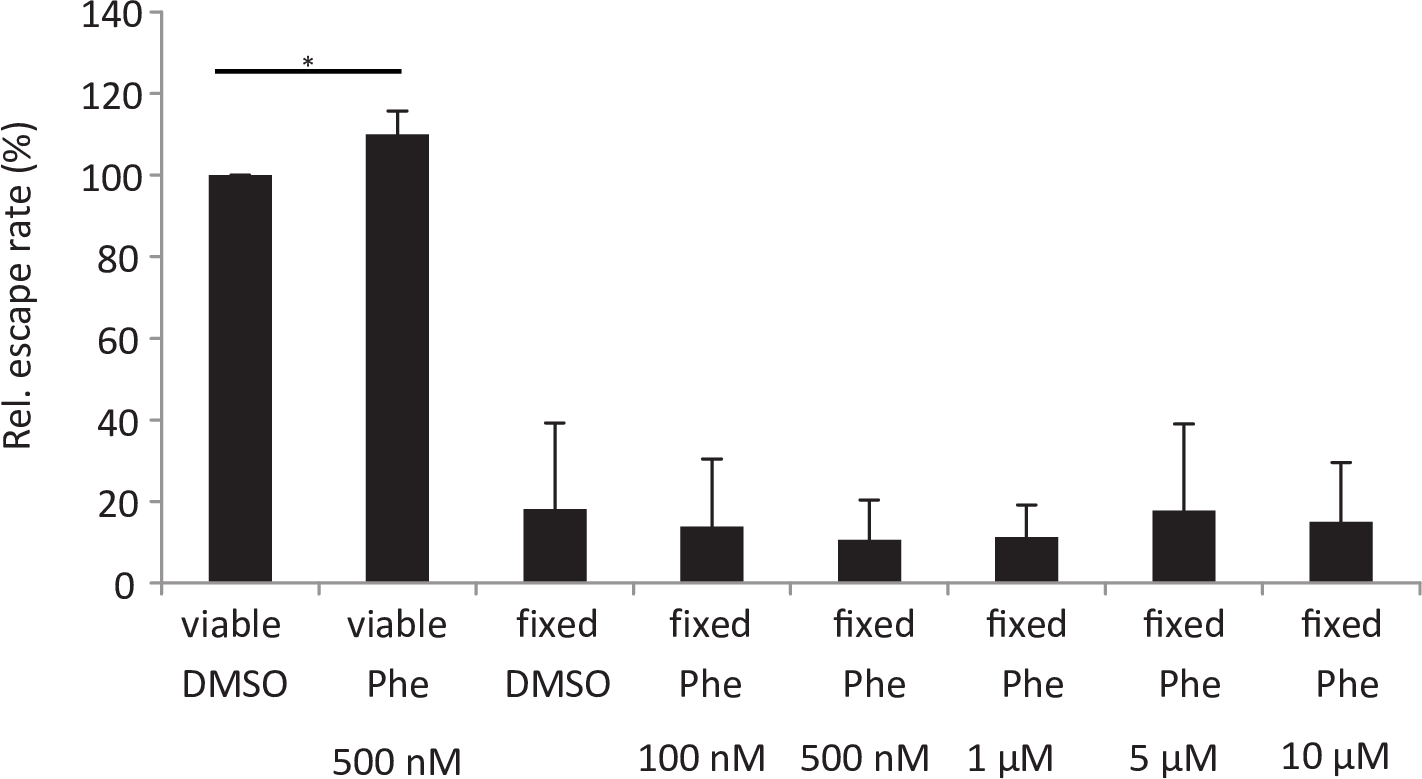

Supplement: S3 Fig — Formaldehyde fixed bacteria were used to infect HeLa YFP-CWT escape reporter cells. Phevalin treatment did not lead to appearance of escape signals, indicating that treatment with the pyrazinones did not alter integrity of endosomal membranes. Bar graphs show the mean of three independent experiments ± SD. Statistical analysis was performed by t-test. (TIF) [file ppat.1005857.s003.tif]

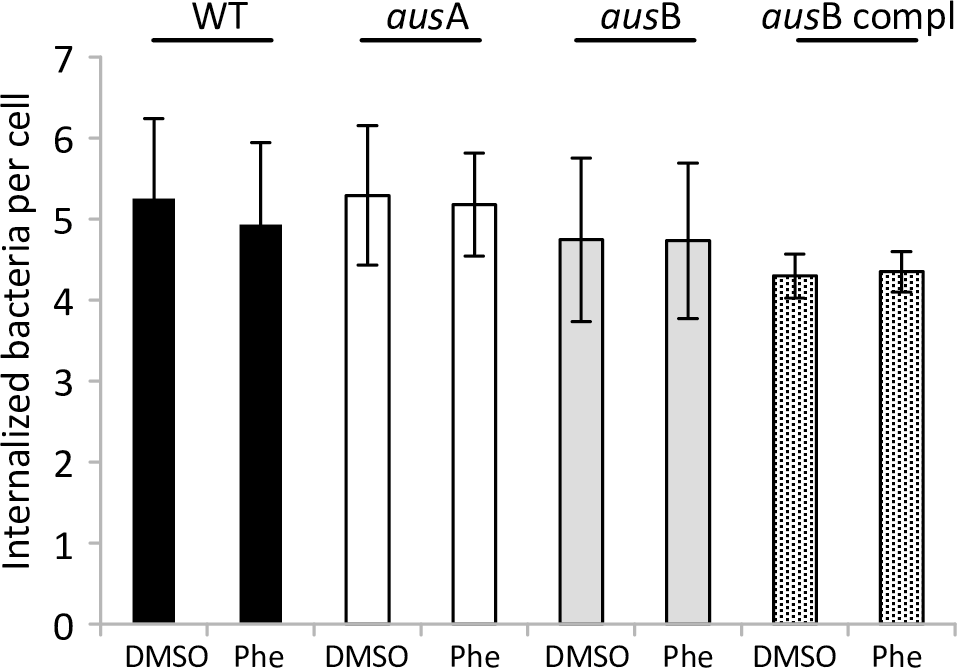

Supplement: S4 Fig — By automated microscopy we enumerated TRITC-labelled intracellular bacteria 3 hours post infection. Wild type S. aureus (WT), mutants within ausA or ausB, as well as the complemented ausB strain (ausB compl) did not show significant changes in intracellular bacteria, regardless of addition of external phevalin to culture medium one hour prior to infection. Bar graphs show the mean of three independent experiments ± SD. Statistical analysis was performed by t-test. (TIF) [file ppat.1005857.s004.tif]

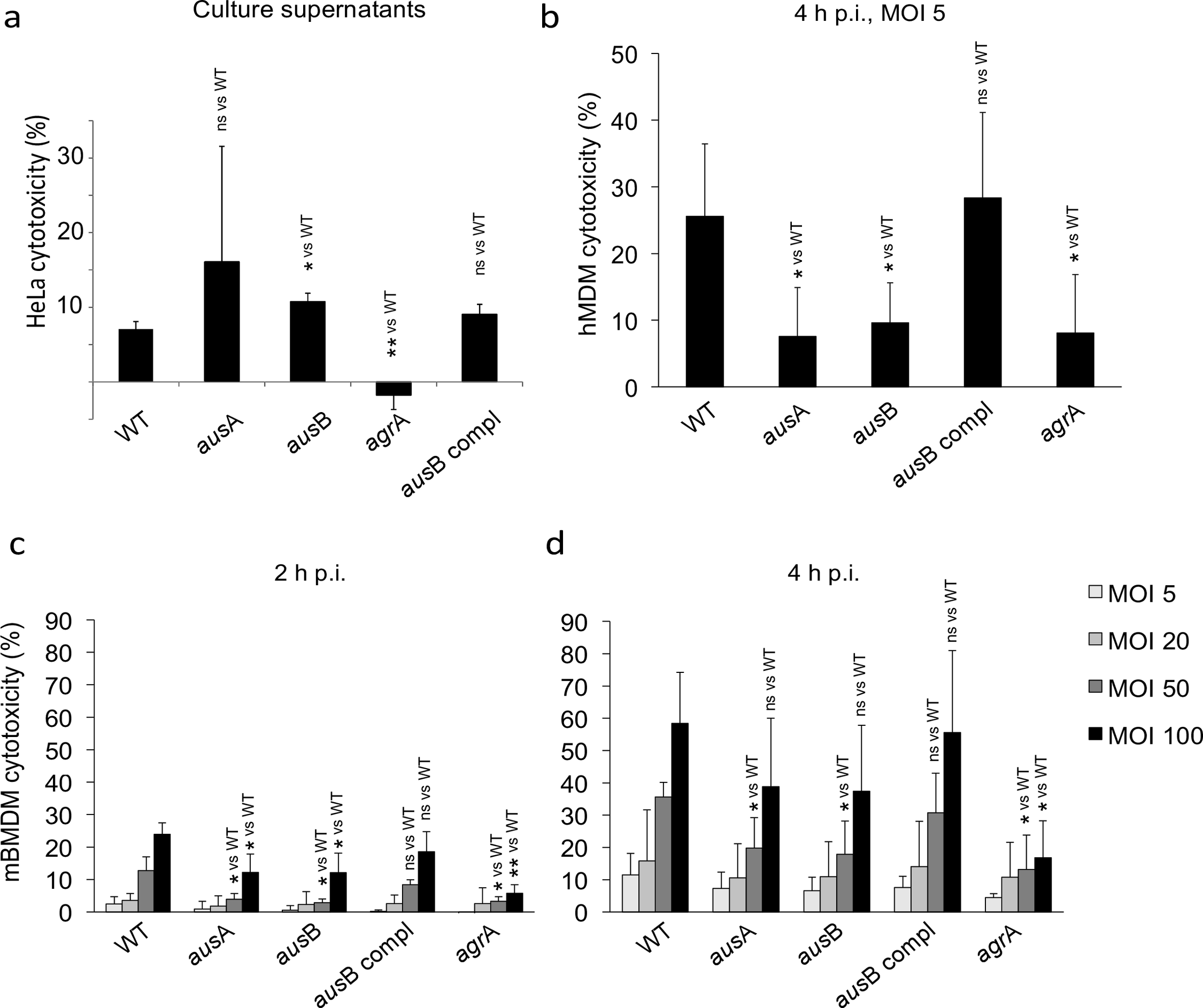

Supplement: S5 Fig — a) Culture supernatants do not exhibit aureusimine-dependent differences in cytotoxicity. We collected culture supernatants of the bacteria and—in addition—of an agrA mutant by centrifugation and sterile filtration and incubated HeLa with a 1:10 dilution of supernatants in RPMI1640 containing 1% FCS. Cell death was measured by LDH release and is plotted as per cent of the positive control. Whereas supernatants of the agrA mutant was apathogenic, no significant changes were observed for the remainder of the strains. b-d) S. aureus cytotoxicity against primary macrophages. Host cell death rates were determined in hMDM 4 hours p.i. (b) and murine bone marrow-derived macrophages (mBMDM) 2 and 4 h p.i. (c,d). Cell death was measured by LDH release as percentage relative to the complete lysate (positive control) and supernatant of uninfected cells (set to 0% cell death, negative control. Graphs show the mean of at least three independent experiments ± SD. Statistical analyses were performed by t-test. *P < 0.05; **P<0.01; ***P < 0.001. (TIF) [file ppat.1005857.s005.tif]

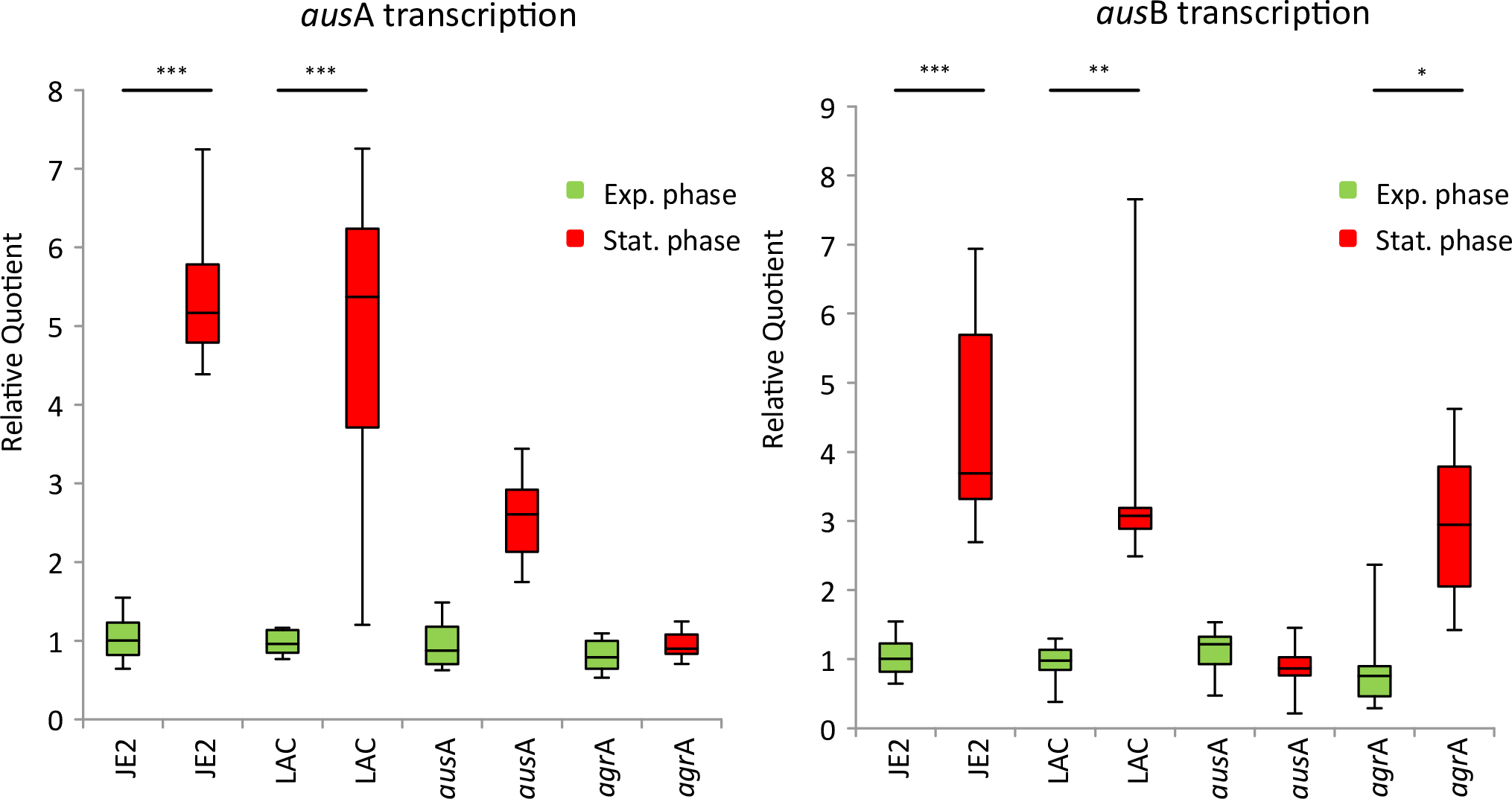

Supplement: S6 Fig — RNA was prepared from S. aureus strain LAC, JE2 as well as the JE2 mutants in ausA or agrA in exponential and stationary growth phases. Transcript abundances of either ausA (left panel) or ausB (right panel) were determined by RT-PCR. Statistical analysis was performed by t-test. *P<0.05; **P<0.01; ***P<0.001. (TIF) [file ppat.1005857.s006.tif]

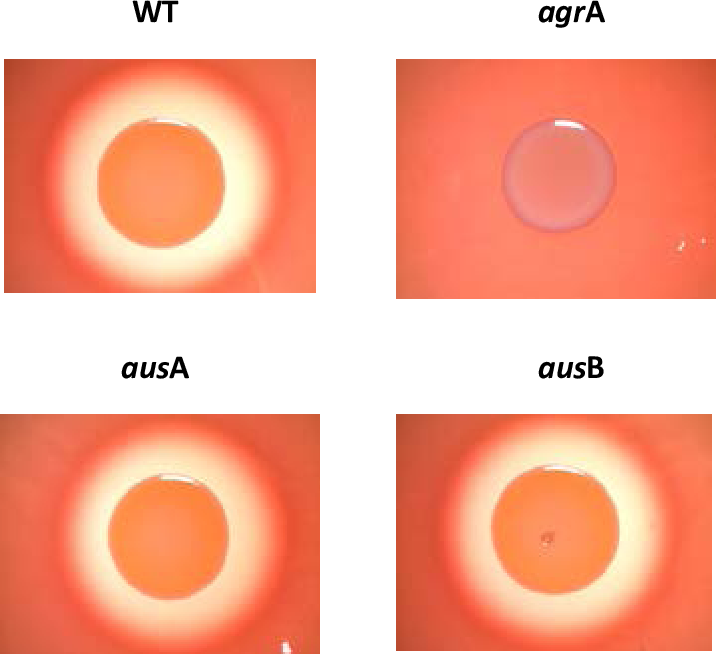

Supplement: S7 Fig — Hemolysis on Columbia blood agar is comparable between S. aureus wild type (WT) and insertional mutants of ausA and ausB, whereas an agrA mutant is non-hemolytic. (TIF) [file ppat.1005857.s007.tif]

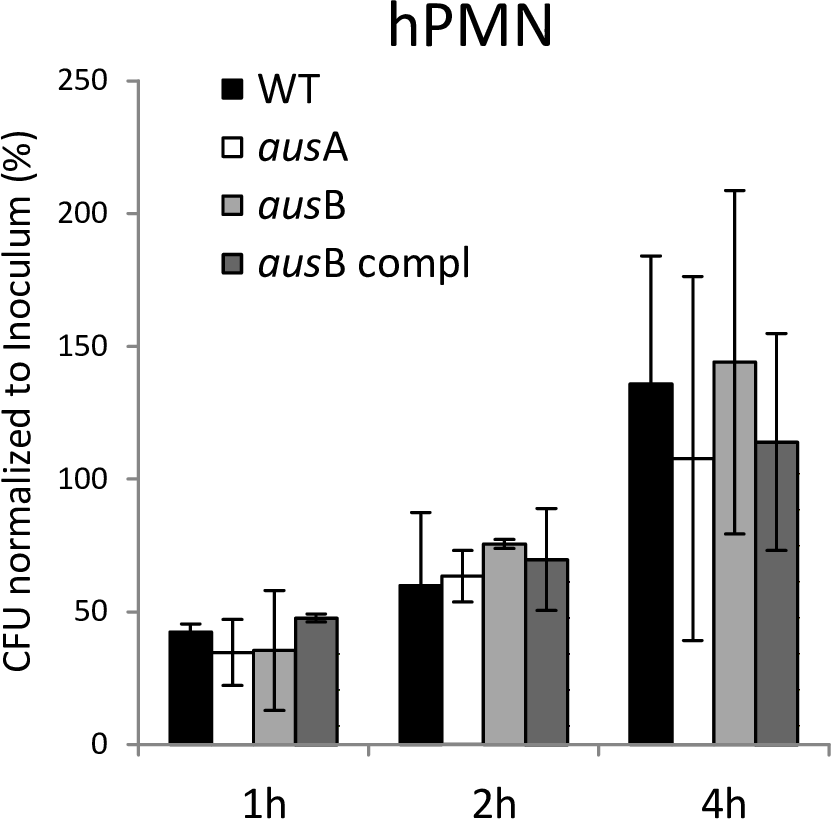

Supplement: S8 Fig — Bacterial replication in neutrophils was determined by CFU recovery assays and was not affected by a mutation in the aus operon. Recovered CFU increased between 1 and 4 hours after infecting PMN with the bacteria regardless of the strain background. (TIF) [file ppat.1005857.s008.tif]

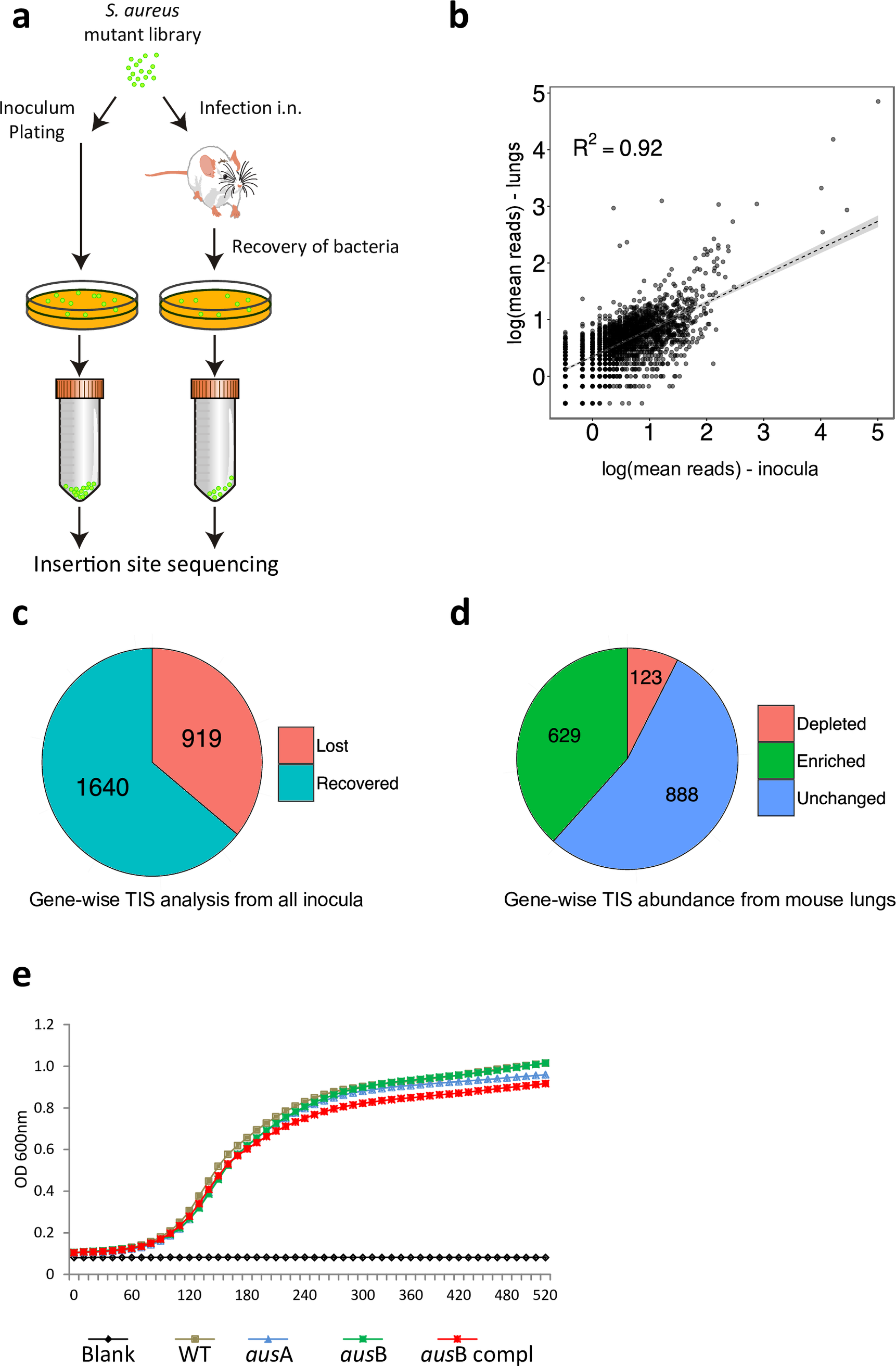

Supplement: S9 Fig — a) Balb/c mice were infected intranasally with a mariner transposon mutant library of S. aureus 6850 comprising ~25,000 mutants. Viable bacteria were recovered from murine lungs 24 h p.i. and pools of recovered bacteria as well as the respective inoculum were analyzed by transposon insertion site sequencing. Changes in transposon insertion sites frequencies were analyzed for mutants enriched or depleted in the pneumonia model. b) Correlation analysis of Tn-seq reads from all TIS depicts high similarity (R2 = 0.92) in composition and complexity between the inocula and mutant library recovered from mouse lungs. c) 1640 out of 2559 genes within S. aureus 6850 were consistently recovered in all inocula used. d) Gene-wise comparison of mutants recovered from mouse lungs with the inocula displays the number of genes that had either depleted, enriched, or unchanged TIS read abundances 24 hours post infection. e) aus mutants do not exhibit growth defects when compared to wild type S. aureus. Triplicates of each culture were used to inoculate 400 μL TSB to OD600 0.1 and were grown up to 20 hours in a 48 micro well plate at 37°C with shaking at 180 rpm. Bacterial growth curves were determined with a TECAN infinite Pro 200 plate reader. Absorbance at 600 nm was recorded in 10 minute intervals. (TIF) [file ppat.1005857.s009.tif]

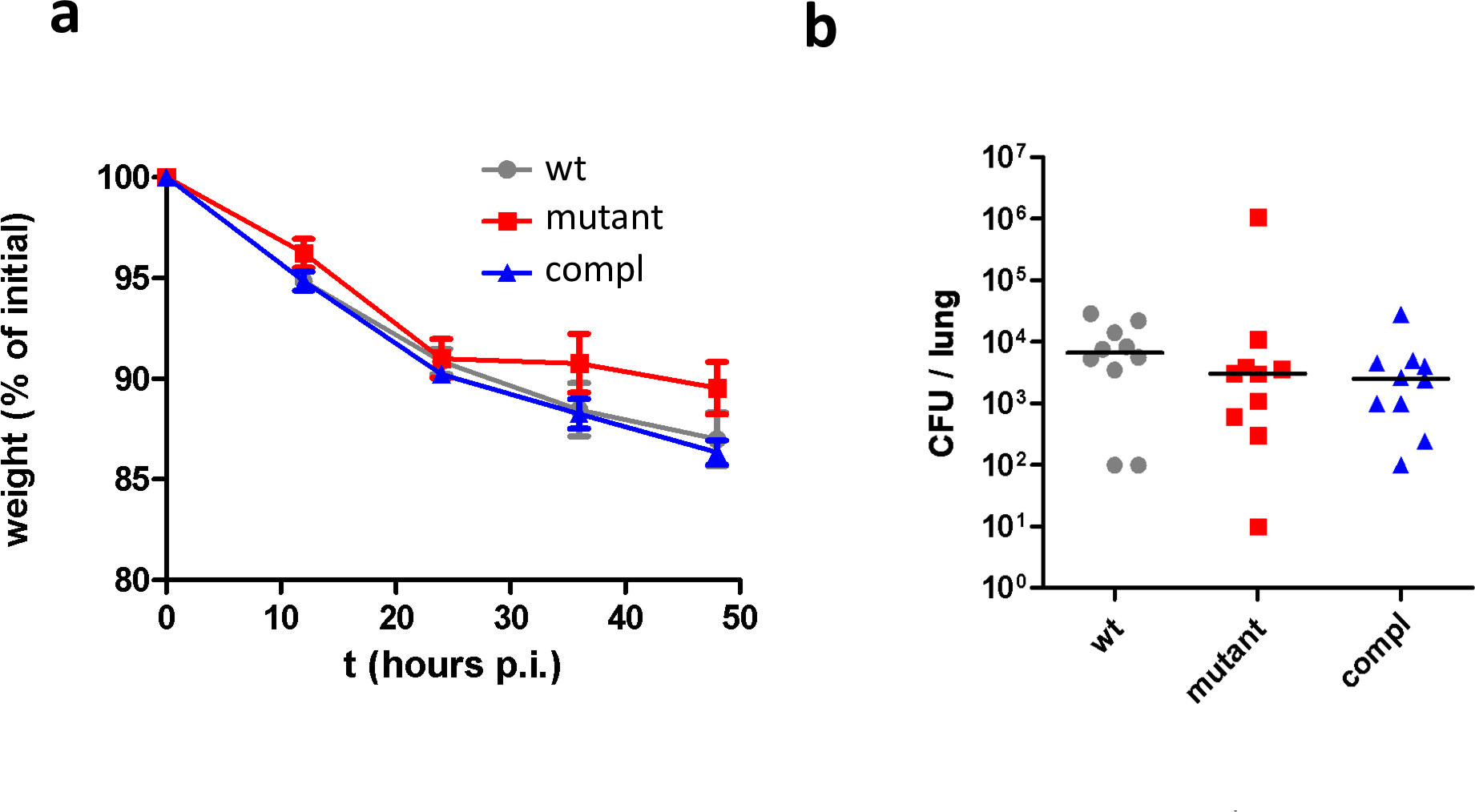

Supplement: S10 Fig — a) The insertional ausB mutant causes less severe disease in the pneumonia model as is indicated by reduced weight loss in mice infected with the mutant. Disease severity is restored upon complementation of ausB in trans (compl.). b) Bacterial CFUs were recovered from lung tissue of infected mice 48 hours p.i. by plating serial dilutions of lysate. Recovered CFUs are not significantly reduced in the mutant when compared to the wild type (wt) or the complemented mutant (compl.). (TIF) [file ppat.1005857.s010.tif]
