# Supplementary material for: Staphylococcus aureus Exploits a Non-ribosomal Cyclic Dipeptide to Modulate Survival within Epithelial Cells and Phagocytes
Source: PLoS Pathog. 2016 Sep 15;12(9):e1005857. doi: 10.1371/journal.ppat.1005857 (PMC5025175; doi:10.1371/journal.ppat.1005857)
Supplement: S1 Table — (PDF) [file ppat.1005857.s011.pdf]

S1 Table : Bacterial Strains used in this study

**Strain**	**Description**	**Source**
Escherichia coli		
DH5α	*fhuA2 lac(del)U169 phoA glnV44 Φ80' lacZ(del)M15 gyrA96 recA1 relA1 endA1 thi-1 hsdR17*	BRL Life Technology
Staphylococcus aureus		
RN4220	NCTC 8325-4 $sau1^-$, $hsdR^-$, laboratory strain accepting foreign DNA; β-toxin producer, no β-hemolysis	[48]
RN4220 p0182	RN4220 with complementation plasmid p0182	This study
6850	methicillin-sensitive; *spa* type t185, sequence type 50 [38], Isolated from a patient with a skin abscess, progressed to bacteremia, osteomyelitis, septic arthritis, and multiple systemic abscesses	[57]
LAC	USA300 CA-MRSA, staphylococcal chromosomal cassette *mec* (SCC*mec*) type IV, *spa*-type 1, Sequence type 8 [ST8].	[53]
LAC Δαβδ	LAC with deletions of the psmα and psmβ operons, as well as a point mutation in the initiation codon of δ-toxin	[54]
JE2	USA300, rsp^+. Derivative of LAC, which was cured of three plasmids.	[18]
JE2 NE119 (*aus*A)	JE2 *aus*A::*bursa*, deficient in aureusimine production	[18]
JE2 NE964 (*aus*B)	JE2 *aus*B::*bursa*, deficient in aureusimine production	[18]
JE2 NE1908 (*pmt*C)	JE2 *pmt*C::*bursa*, mutant in the phenol-soluble modulin transporter component, *pmt*C	[18]
JE2 NE1532 (*agr*A)	JE2 *agr*A::*bursa*, non-hemolytic insertional mutant in the quorum sensing system *agr*	[18]
NE964 p0182	NE964 carrying plasmid p0182, aureusimine producer	This study
