# Supplementary material for: Staphylococcus aureus Exploits a Non-ribosomal Cyclic Dipeptide to Modulate Survival within Epithelial Cells and Phagocytes
Source: PLoS Pathog. 2016 Sep 15;12(9):e1005857. doi: 10.1371/journal.ppat.1005857 (PMC5025175; doi:10.1371/journal.ppat.1005857)
Supplement: S2 Table — (PDF) [file ppat.1005857.s012.pdf]

S2 Table: Plasmids used in the study

**Name**	**Description**	**Source**
pBTn	plasmid for transposon mutagenesis	[58]
p0182	*aus*B complementation plasmid	This study
