# Supplementary material for: Staphylococcus aureus Exploits a Non-ribosomal Cyclic Dipeptide to Modulate Survival within Epithelial Cells and Phagocytes
Source: PLoS Pathog. 2016 Sep 15;12(9):e1005857. doi: 10.1371/journal.ppat.1005857 (PMC5025175; doi:10.1371/journal.ppat.1005857)
Supplement: S3 Table — (PDF) [file ppat.1005857.s013.pdf]

S3 Table: PCR and Adaptor Oligonucleotides

Name	Sequence (5‘-3‘)
**TIS Sequencing**	
MultiPlex-Y-Adapt_r	ACACTCTTTCCCTACACGACGCTCTTCCGATC*T
MultiPlex-Y-Adapt_f	[Phos]GATCGGAAGAGCACACGTCT
Himar_TnSeq_Read1_v2	ACCGAGATCTACGGACTTATCAGCCAACCTGT
MP-TnSeq_Index2 (barcode CGATGT)	CAAGCAGAAGACGGCATACGAGATACATCGGTGACTGGAGTTCAGACGTGTGCTCTTCCGATCT
MP-TnSeq_Index7 (barcode CAGATC)	CAAGCAGAAGACGGCATACGAGATGATCTGGTGACTGGAGTTCAGACGTGTGCTCTTCCGATCT
MP-TnSeq_Index8 (barcode ACTTGA)	CAAGCAGAAGACGGCATACGAGATTCAAGTGTGACTGGAGTTCAGACGTGTGCTCTTCCGATCT
MP-TnSeq_Index9 (barcode GATCAG)	CAAGCAGAAGACGGCATACGAGATCTGATCGTGACTGGAGTTCAGACGTGTGCTCTTCCGATCT
MP-TnSeq_Index10 (barcode TAGCTT)	CAAGCAGAAGACGGCATACGAGATAAGCTAGTGACTGGAGTTCAGACGTGTGCTCTTCCGATCT
TnSeq-HimarPCR_v2-PT	GACCACCGAGATCTACGAGACCGGGGACTTATCAGC
himar-5-PCR	CCATAACTTTAGGGTTAACCATACGC
himar-3-PCR	CAGCTTCCAAGGAGCTAAAGAGGTCC
IS-Himar-forward	AATGATACGGCGACCACCGAGATCT
IS-BC-Reverse	CAAGCAGAAGACGGCATACGAGAT
**RT-PCR**	
RT-*gyr*B-R	ATAGCCTGCTTCAATTAACG
RT-*gyr*B-F	CGACTTTGATCTAGCGAAAG
0181_for_RT	GTGTCCATTTTCGATGATAGTC
0181_rev_RT	TGTCAGAGTTATTCTCTAACCG
0182_for_RT	AACCGCGTACAGTCAACTATAG
0182_rev_RT	ATAAGCTCACATAGATGGGC
**Cloning**	
0182_for	CATCCTAGGAGGAAAGTTATGACAGTATTTGTAATGC
0182_rev	GACCCTAGGTTAACTACTCAATAACTGAAATACAGACAC
SarAP1-R	GGTACCGATGCATCTTGCTCGATACATTTG
SarAP1-F	GTCGACGCGGCCGCTGCATGCCTGATATTTTTG

*indicates a phosphothioate bond; [Phos] indicates 5' phosphorylation; underlined sequences of index primers indicate barcode sequences
