# Supplementary material for: Staphylococcus aureus Exploits a Non-ribosomal Cyclic Dipeptide to Modulate Survival within Epithelial Cells and Phagocytes
Source: PLoS Pathog. 2016 Sep 15;12(9):e1005857. doi: 10.1371/journal.ppat.1005857 (PMC5025175; doi:10.1371/journal.ppat.1005857)
Supplement: S4 Table — (PDF) [file ppat.1005857.s014.pdf]

S4 Table: Escape efficiencies of tested S. aureus mutants

Library label	Gene Description	Identifier	n	% escape[1]	Std Dev
NE4	ABC transporter ATP-binding protein	SAUSA300_0309	1	90.2	-
NE10	putative hemolysin III	SAUSA300_2129	1	75.8	-
NE12	drug resistance transporter, EmrB/QacA subfamily	SAUSA300_2126	1	84.6	-
NE15	transcriptional regulator, TetR family	SAUSA300_2509	1	89.6	-
NE17	putative drug transporter	SAUSA300_1705	1	90.4	-
NE26	*coa*; staphylocoagulase precursor	SAUSA300_0224	2	116.8	22.1
NE30	hydrolase, haloacid dehalogenase-like family	SAUSA300_0600	1	81.4	-
NE32	transcriptional regulator, LysR family domain protein	SAUSA300_0095	1	104.9	-
NE49	DNA-binding response regulator, AraC family	SAUSA300_0217	2	92.4	16.6
NE67	*isa*B; immunodominant antigen B	SAUSA300_2573	1	106.0	-
NE75	Cap1B; capsular polysaccharide biosynthesis protein Cap1B	SAUSA300_2597	1	107.8	-
NE91	*kdp*A; K+-transporting ATPase, A subunit	SAUSA300_2034	1	113.6	-
NE95	*agr*B; accessory gene regulator protein B	SAUSA300_1989	3	15.8	1.4
NE96	*sar*U; staphylococcal accessory regulator U	SAUSA300_2438	3	88.4	18.9
NE99	transcriptional regulator, Fur family	SAUSA300_1448	1	121.5	-
NE100	FtsK/SpoIIIE family protein	SAUSA300_1482	1	105.2	-
NE104	putative lipase/esterase	SAUSA300_0641	3	78.2	29.1
NE110	transcriptional regulator, GntRfamily/aminotransferase, class I	SAUSA300_0110	1	102.1	-
NE111	putative surface protein	SAUSA300_0883	1	110.8	-
NE116	putative sensor histidine kinase	SAUSA300_1219	1	116.6	-
NE119	non-ribosomal peptide synthetase	SAUSA300_0181	4	47.3	12.4
NE129	DNA internalization-related competence proteinComEC/Rec2	SAUSA300_1547	1	100.5	-
NE132	putative transcriptional regulator	SAUSA300_2640	1	110.0	-
NE138	putative membrane protein	SAUSA300_0275	1	97.0	-
NE147	sensor histidine kinase	SAUSA300_0254	1	111.7	-
NE163	*aur*; zinc metalloproteinase aureolysin	SAUSA300_2572	2	72.6	27.5
NE165	staphylococcal accessory regulator	SAUSA300_0114	1	86.0	-
NE179	multidrug resistance protein	SAUSA300_2360	1	92.8	-
NE185	siderophore biosynthesis protein, IucA/IucC family	SAUSA300_0122	1	81.2	-
NE186	*fnb*A; fibronectin binding protein A	SAUSA300_2441	1	68.6	-

NE210	staphylococcal accessory regulator	SAUSA300_2247	1	81.3	-
NE212	putative lysophospholipase	SAUSA300_1710	1	96.5	-
NE214	siderophore biosynthesis protein, IucC family"	SAUSA300_0123	1	89.0	-
NE217	*pkn*B; protein kinase	SAUSA300_1113	1	80.6	-
NE218	sensor histidine kinase family protein	SAUSA300_0218	2	100.0	12.3
NE251	polysaccharide biosynthesis protein	SAUSA300_0482	1	83.8	-
NE258	*cls*; cardiolipin synthetase	SAUSA300_2044	3	85.0	35.7
NE262	DNA-binding response regulator, LuxR family	SAUSA300_1220	2	81.9	9.0
NE274	conserved hypothetical protein	SAUSA300_1867	1	82.3	-
NE281	transcriptional regulator, MarR family	SAUSA300_2452	1	69.0	-
NE286	immunoglobulin G binding protein A precursor	SAUSA300_0113	2	127.1	37.6
NE287	conserved hypothetical protein	SAUSA300_1744	2	82.0	23.9
NE290	transferrin receptor	SAUSA300_0721	2	87.6	0.9
NE294	signal transduction protein TRAP	SAUSA300_1784	3	79.1	14.1
NE296	staphylococcal accessory protein X	SAUSA300_0654	1	83.8	-
NE324	regulatory protein RecX	SAUSA300_1854	2	91.9	15.5
NE331	exonuclease RexA	SAUSA300_0870	2	71.0	16.1
NE332	iron transport associated domain protein	SAUSA300_1029	2	102.1	8.6
NE334	Hyaluronate lyase precursor	SAUSA300_2161	1	73.7	-
NE338	*lip*, triacylglycerol lipase precursor	SAUSA300_2603	1	87.8	-
NE354	transcriptional regulator, gntR family protein	SAUSA300_0503	2	104.2	11.4
NE355	transcriptional regulator, TetR family	SAUSA300_2515	1	108.3	-
NE362	putative teichoic acid biosynthesis protein B	SAUSA300_0247	2	100.9	11.3
NE367	arginine repressor	SAUSA300_2571	2	95.4	0.9
NE369	*gua*C, guanosine monophosphate reductase	SAUSA300_1235	1	105.5	-
NE374	*feo*B, ferrous iron transport protein B	SAUSA300_2487	2	119.2	22.7
NE383	Putative membrane protein	SAUSA300_0279	2	105.5	15.7
NE386	*rot*, staphylococcal accessory regulator Rot	SAUSA300_1708	2	76.5	26.4
NE391	*clf*B, clumping factor B	SAUSA300_2565	1	106.5	-
NE392	peptide ABC transporter, ATP-binding protein	SAUSA300_0200	2	90.1	10.0
NE399	preprotein translocase, SecY protein	SAUSA300_2588	3	100.8	27.3
NE400	iron compound ABC transporter, iron compound-binding protein	SAUSA300_2136	3	79.8	26.0
NE406	*fhu*A, errichrome transport ATP-binding protein fhuA	SAUSA300_0633	1	112.3	-
NE410	*ure*C, urease, alpha subunit	SAUSA300_2240	2	96.4	16.2

NE415	putative transcriptional regulator	SAUSA300_2259	4	96.5	30.1
NE420	Pbp3, penicillin-binding protein 3	SAUSA300_1512	1	105.8	-
NE426	*tat*C, Sec-independent protein translocase TatC	SAUSA300_0347	1	87.6	-
NE431	iron-dependent repressor	SAUSA300_0621	4	92.7	24.6
NE432	*sdr*C protein	SAUSA300_0546	2	104.2	28.3
NE434	phosphosugar-binding transcriptional regulator	SAUSA300_2271	2	100.6	26.9
NE444	aminopeptidase PepS	SAUSA300_1860	2	106.5	5.2
NE447	putative iron-sulfur cluster-binding protein	SAUSA300_1806	2	112.8	19.1
NE453	Sbi, IgG-binding protein SBI	SAUSA300_2364	1	119.4	-
NE454	*arc*R, transcriptional regulator, Crp/Fnr family	SAUSA300_2566	2	105.9	28.6
NE457	Peptide ABC transporter, permease protein	SAUSA300_0202	2	122.7	0.3
NE462	putative membrane protein	SAUSA300_0917	2	111.5	23.4
NE481	DNA-binding response regulator	SAUSA300_0645	2	96.9	23.6
NE483	*pep*T, peptidase T	SAUSA300_0727	2	102.0	28.5
NE505	transcriptional regulator, LysR family domain protein	SAUSA300_0093	2	108.5	11.4
NE512	modC, molybdenum ABC transporter, ATP-binding protein ModC	SAUSA300_2228	2	122.0	12.4
NE513	putative helicase	SAUSA300_2431	2	88.0	0.2
NE514	*sar*T, staphylococcal accessory regulator T	SAUSA300_2437	4	104.8	13.9
NE519	peptidase, M20/M25/M40 family	SAUSA300_1460	2	95.5	11.6
NE528	putative permease	SAUSA300_0091	2	97.7	11.8
NE530	transcriptional regulator, AraC family	SAUSA300_2248	2	85.7	26.7
NE535	*sps*A, signal peptidase IA	SAUSA300_0867	3	48.8	11.5
NE539	*fad*E, acyl-CoA synthetase FadE	SAUSA300_0228	3	92.2	18.2
NE541	peptide ABC transporter, permease protein	SAUSA300_0201	1	106.7	-
NE551	putative fibronectin/fibrinogen binding protein	SAUSA300_1101	1	108.4	-
NE554	*vra*R, DNA-binding response regulator	SAUSA300_1865	1	100.8	-
NE557	iron transport associated domain protein	SAUSA300_1030	1	97.8	-
NE558	*luk*E, leukotoxin LukE	SAUSA300_1769	3	105.3	27.5
NE567	transcriptional regulator, MarR family	SAUSA300_2331	2	98.6	28.2
NE571	putative transposase	SAUSA300_2263	1	87.0	-
NE578	iron compound ABC transporter, permease protein	SAUSA300_0599	1	77.2	-
NE584	*nar*K, nitrite extrusion protein	SAUSA300_2333	2	122.7	46.6
NE588	*srr*B, staphylococcal respiratory response protein, *srr*B	SAUSA300_1441	2	88.4	42.5

NE600	putative peptidase	SAUSA300_2087	2	96.9	10.0
NE610	*lys*P, lysine-specific permease	SAUSA300_1628	2	89.9	5.2
NE618	*pho*R, sensory box histidine kinase PhoR	SAUSA300_1638	2	92.7	17.7
NE636	exfoliative toxin A	SAUSA300_1065	1	99.6	-
NE637	Cbf1, cmp-binding-factor 1	SAUSA300_1791	2	115.0	8.7
NE643	staphylococcal tandem lipoprotein	SAUSA300_0102	2	110.1	15.1
NE647	conserved hypothetical protein	SAUSA300_1485	1	95.8	-
NE648	*glv*C, PTS system, arbutin-like IIBC component	SAUSA300_2270	2	73.5	2.8
NE672	homoserine kinase	SAUSA300_1228	1	113.9	-
NE679	Pbp4, penicillin-binding protein 4	SAUSA300_0629	1	100.4	-
NE773	drug transporter	SAUSA300_2451	1	79.3	-
NE873	*agr*C, accessory gene regulator protein C	SAUSA300_1991	3	13.2	8.8
NE934	cysteine protease precursor	SAUSA300_0950	1	77.0	-
NE964	4'-phosphopantetheinyl transferase superfamily protein	SAUSA300_0182	4	52.4	11.7
NE993	Putative hemolysin III	SAUSA300_2129	1	86.7	-
NE1065	osmoprotectant ABC transporter permease	SAUSA300_0707	1	76.5	-
NE1077	*rbs*K, Ribokinase	SAUSA300_0262	3	74.7	18.1
NE1109	*rpo*F, RNA polymerase sigma factor SigB	SAUSA300_2022	3	98.3	15.0
NE1181	putative membrane protein	SAUSA300_1912	1	92.6	-
NE1188	putative membrane protein	SAUSA300_1912	3	101.9	61.5
NE1193	*sar*A, accessory regulator A	SAUSA300_0605	5	94.9	18.0
NE1224	Fe/Mn family superoxide dismutase	SAUSA300_0135	1	91.2	-
NE1234	*lct*P, L-lactate permease	SAUSA300_0112	3	86.6	30.9
NE1240	*luk*D, leukotoxin LukD	SAUSA300_1768	1	101.2	-
NE1296	*sae*S, sensor histidine kinase SaeS	SAUSA300_0690	5	66.0	9.9
NE1304	*rsp*, transcription regulatory protein	SAUSA300_2326	5	82.3	7.9
NE1354	Alpha-hemolysin precursor	SAUSA300_1058	3	85.3	23.1
NE1363	*srt*B, sortase B	SAUSA300_1034	1	109.6	-
NE1366	catalase	SAUSA300_1232	2	99.9	27.1
NE1386	*luk*A, Leukocidin/Hemolysin toxin family protein	SAUSA300_1974	4	35.9	13.7
NE1399	*hlg*A, gamma-hemolysin component A	SAUSA300_2365	1	80.4	-
NE1449	*hlg*C, gamma-hemolysin component C	SAUSA300_2366	1	89.4	-
NE1472	*rsb*W, anti-sigma-B factor, serine-protein kinase	SAUSA300_2023	3	25.6	9.2

NE1532	*agr*A, accessory gene regulator protein A	SAUSA300_1992	5	6.4	4.4
NE1555	*cod*Y, transcriptional repressor CodY	SAUSA300_1148	4	92.2	12.2
NE1607	*rsb*U, sigma-B regulation protein	SAUSA300_2025	3	53.3	24.8
NE1663	diacylglycerol glucosyltransferase	SAUSA300_0918	4	93.3	12.3
NE1682	*hlg*B, gamma-hemolysin component B	SAUSA300_2367	1	104.0	-
NE1714	GTP pyrophosphokinase	SAUSA300_1590	3	74.7	6.5
NE1756	sensor histidine kinase	SAUSA300_0646	1	86.5	-
NE1775	triacylglycerol lipase	SAUSA300_0320	1	95.9	-
NE1787	*srt*A, sortase	SAUSA300_2467	1	112.3	-
NE1848	*luk*S-PV, Panton-Valentine leukocidin, LukS-PV	SAUSA300_1382	3	133.8	23.9
NE1868	*mec*A, penicillin-binding protein 2'	SAUSA300_0032	1	106.2	-
NE1872	*rsb*V, anti-sigma-B factor, antagonist	SAUSA300_2024	2	102.5	68.1
NE1875	truncated beta-hemolysin	SAUSA300_1918	2	118.3	38.1
NE1908	ABC transporter ATP-binding protein	SAUSA300_1911	3	29.3	18.3

[1] Normalized to the phagosomal escape rate of the USA300 JE2 wild type detected in the specific experiment. If n >1 then a mean value of all normalized escape rates is shown.
