# Supplementary material for: Staphylococcus aureus Exploits a Non-ribosomal Cyclic Dipeptide to Modulate Survival within Epithelial Cells and Phagocytes
Source: PLoS Pathog. 2016 Sep 15;12(9):e1005857. doi: 10.1371/journal.ppat.1005857 (PMC5025175; doi:10.1371/journal.ppat.1005857)
Supplement: S5 Table — (PDF) [file ppat.1005857.s015.pdf]

S5 Table: S. aureus transposon mutants that were significantly depleted or enriched in a murine pneumonia model

**locus**	**log2 FC**	**p-val.**	**adj. p-val.**	**product**	**gene**
RSAU_000335	-4.89	0.000	0.000	bifunctional GMP synthase/glutamine amidotransferase protein	*gua*A
RSAU_002354	-3.99	0.004	0.090	hypothetical protein	
RSAU_000347	-3.97	0.004	0.090	hypothetical protein	
RSAU_000919	-3.97	0.004	0.090	hypothetical protein	
RSAU_001762	-3.82	0.000	0.011	type 1 glutamine amidotransferase (GATase1)-like protein	
RSAU_000524	-3.76	0.010	0.093	hexulose-6-phosphate synthase, putative	*hps*
RSAU_002139	-3.69	0.005	0.090	hypothetical protein	
RSAU_002284	-3.66	0.004	0.090	major facilitator superfamily drug transporter, putative	
RSAU_000898	-3.58	0.006	0.090	hypothetical protein	
RSAU_002181	-3.48	0.000	0.011	CorA-like Mg2 transporter protein	
RSAU_000034	-3.37	0.007	0.090	putative transposase	
RSAU_001071	-3.12	0.010	0.093	YlmG membrane protein	*ylm*G
RSAU_002127	-3.12	0.008	0.090	urease accessory protein UreD, putative	*ure*D
RSAU_000072	-2.77	0.009	0.093	IucC family siderophore biosynthesis protein	*sbn*C
RSAU_000130	-2.15	0.008	0.090	non-ribosomal peptide synthetase, putative	*aus*A
RSAU_003007	-2.05	0.010	0.093	ssr42	ssr42
RSAU_000802	3.30	0.007	0.090	5'-nucleotidase, putative	
RSAU_001966	3.53	0.008	0.090	DNA-directed RNA polymerase, delta subunit	*rpo*E
RSAU_000703	4.10	0.007	0.090	7-cyano-7-deazaguanine reductase	*que*F
