# Supplementary material for: Staphylococcus aureus Exploits a Non-ribosomal Cyclic Dipeptide to Modulate Survival within Epithelial Cells and Phagocytes
Source: PLoS Pathog. 2016 Sep 15;12(9):e1005857. doi: 10.1371/journal.ppat.1005857 (PMC5025175; doi:10.1371/journal.ppat.1005857)
Supplement: S6 Table — (PDF) [file ppat.1005857.s016.pdf]

S6 Table: Clinical score determination of mice to assess severity of disease

Observation	Score points
**I Body weight**	
- No change	0
- Loss of body weight in % = score points; e.g. loss of body weight 8% = 8 points	1-20
- Loss of body weight ≥20%	20
**II General conditions**	
**Fur**	
- Shining	0
- Matte	2
- Ruffled	4
**Eyes**	
- Clear and clean	0
- Unclean and sticky, closed or semi-closed	3
**Posture**	
- Normal	0
- Hunched	10
- Massively hunched	20
**Clinical complications**	
- Tension, paralysis, tremor	20
- Breath noises	20
- Animal feels cold to the touch	20
**III Motility**	
- Spontaneous (normal behavior, social contacts)	0
- Spontaneous but reduced	1
- Moderately reduced activity	2
- Motility only after stimulation	5
- Isolation, lethargy, coordination disorders	10
- Self-mutilation, aggression	20
**IV Respiration**	
- Breathing normal	0
- Breathing slightly changed	1
- Accelerated breathing + 30% (tachypnoea)	10
- Strongly accelerated breathing + 50%	20

Rating	measures	Sum score pts
Severity level 0	no burden on animals, animals healthy	0-3
Severity level 1	low burden on animals, low sickness, animals are observed	4-9
Severity level 2	moderate burden on animals, moderate sickness, animals are carefully observed	10-15
Severity level 3	moderate to severe burden on animals, moderate to severe sickness, animals are carefully observed, abort of experiment if necessary	16-20
Severity level 4	severe burden on animals, animals moribund, implementation of humane endpoint, abort of experiment	>20
